# Supplementary material for: Control of CRK-RAC1 activity by the miR-1/206/133 miRNA family is essential for neuromuscular junction function
Source: Nat Commun. 2022 Jun 8;13:3180. doi: 10.1038/s41467-022-30778-7 (PMC9178026; doi:10.1038/s41467-022-30778-7)
Supplement: Supplementary file 1 — Supplementary Information [file 41467_2022_30778_MOESM1_ESM.pdf]

Supplementary Information

Control of CRK-RAC1 activity by the *miR-1/206/133* miRNA family is essential for neuromuscular junction function

Ina Klockner, Christian Schutt, Theresa Gerhardt, Thomas Boettger*, Thomas Braun*

Max Planck Institute for Heart- and Lung Research, Department of Cardiac Development and Remodelling, Ludwigstr. 43, D-61231 Bad Nauheim, Germany: Phone +49 6032 7051115 Fax +49 6032 7051104

*Corresponding authors: Thomas Boettger and Thomas Braun; thomas.boettger@mpi-bn.mpg.de, thomas.braun@mpi-bn.mpg.de

**Supplementary Information**	**Page**
Supplementary Methods	3
Supplementary Table 1	7
Supplementary Table 2	8
Supplementary Table 3	10
Supplementary Figure 1	11
Supplementary Figure 2	13
Supplementary Figure 3	14
Supplementary Figure 4	16
Supplementary Figure 5	18
Supplementary Figure 6	20
Supplementary Figure 7	22
Supplementary Figure 8	23
Supplementary Figure 9	24
Supplementary Figure 10	25
Supplementary Figure 11	26
Supplementary Figure 12	27
Supplementary Figure 13	28
Supplementary Figure 14	30
References	31

Supplementary Methods

Immunoprecipitation

Protein extracts were prepared using limb muscle tissue from *miR-1/206/133* tKO and WT pups at E18.5 in RIPA buffer (50 mM Tris-HCl pH7.4, 150 mM NaCl, 1% N-P40, 0.25% Na-Deoxycholate, one complete Protease Inhibitor cocktail tablet, Roche #4693132001) by sonication (Bandelin Sonoplus) using cycle 5, 30% power for 30 seconds. Muscle lysates were incubated at 4 °C on a rotating wheel for 30 min, followed by centrifugation for 20 min at 21000 g and 4 °C. Supernatants were collected and stored at -80 °C until further use. 60 µl per sample G-Sepharose-beads (Millipore #P3296) were washed two times using RIPA buffer, followed by a pre-blocking step for 1 h in 3% BSA (Sigma #A7284) in RIPA buffer at 4 °C on a rotating wheel for 1 hour. Next, the beads were washed twice with RIPA buffer and re-suspended in 30 µl RIPA buffer. 500 µg protein lysate in 300 µl per sample were incubated with 30 µl bead suspension to prevent non-specific protein binding to the beads at 4 °C on a rotating wheel for 1 h. The supernatants were collected and 4 µg of anti-CRK (Santa Cruz #sc-390132) was added. 30 µl of pre-blocked bead suspension were also added followed by an incubation at 4 °C on a rotating wheel overnight. At the next day, beads were washed three times using RIPA buffer at 4 °C on a rotating wheel for 10 min. Co-precipitated proteins were identified by western blot analysis or mass spectrometry (MS), using equal amounts of samples for analysis.

Western blot analysis and immunofluorescence staining

Skeletal muscle was lysed in extraction buffer (0.1 M Tris/HCl (pH 8.0), 0.01 M EDTA (pH 8.0), 10% SDS, 5% DTT, 1 mM Na_3VO_4, 20 mM NaF, complete Protease Inhibitor cocktail, Roche #11697498001) by sonication (Bandelin Sonoplus) using cycle 5, 30% power for 30 seconds. 10-40 µg of protein extracts were separated on a 4-20% gradient SDS-PAGE (ThermoFischer #NP0321BOX) and blotted onto nitrocellulose membranes. Blocking of membranes was performed with either 3% BSA or 5% milk powder in TBS-T. The following antibodies were used to probe the blots: anti-CRK (1:1000, ~42 kDa, Santa Cruz #sc-390132/ BDBiosciences #610035), anti-FARP1 (~119 kDa, 1:5000, ThermoFisher #PA5-99105), anti-GAPDH (~37 kDa, 1:1000, CST #2118), anti-HA-tag to detect dCas9-SPH (1:1000, ~115 kDa, Covance #MMS-101P), anti-MEF2A (~54 kDa, 1:1000, CST #9736), PAK1/2/3 (~61 kDa PAK2, ~68 kDa PAK1/3, 1:1000, CST #2604), Phospho-PAK1/2/3 (61-67 kDa PAK2, 68-74 kDa PAK1/3, 1:1000, CST #2606), anti-RAC1 (1:1000, ~22 kDa, part of kit, ThermoFisher #16118) and anti-RALA (1:1000, ~26 kDa, BD Bioscience s#610222). The following secondary antibodies were used: anti-mouse-HRP (1:5000, ThermoFischer #31450) and anti-rabbit-HRP (1:5000, ThermoFischer #31460). Quantification of western blots was performed by densitometric scanning after signal visualization with the SuperSignal Pico plus kit (ThermoFisher #34578) and Versadoc-System (Biorad).

Cultured cells, isolated myofibers and cryosections were mounted on Superfrost slides (ThermoFisher #J3800AMNZ) and used for immunofluorescence staining. Samples were fixed for 7 min with 4% paraformaldehyde in PBS and washed three times 10 min using 0.3% Triton X-100/PBS. Blocking was performed for 1 h in blocking solution (0.01% Triton X-100, 1/10 Blocking One (Nacalai#03953-95)/PBS). When using antibodies produced in mice, Mouse-on-Mouse Blocking Reagent (Vector labs#MKB-2213) was substituted 1:25. After a 10 min wash step in PBS, the sections were incubated with primary antibodies overnight at 4 °C. Subsequently, the sections were washed using 0.01% Triton X-100/PBS as described above. The Incubation of the secondary antibodies and BTX was performed at room temperature for 1 h, followed by two washing steps in 0.01% Triton X-100/PBS. DAPI was diluted in 0.01% Triton X-100/PBS, incubated for 5 min. After a final washing step the samples were embedded using Fluoromount W (Serva #21634.01). The following primary and secondary antibodies were used: rabbit anti-Laminin (1:1000, Sigma #L9393), mouse anit-CRK (1:1000, BDBiosciences #610035), rabbit anti-GFP (1:1000, ThermoFisher #A-11122), goat anti-mouse IgG-Alexa Fluor488 (1:1000, Jackson #115-545-205), goat anti-mouse IgG-Alexa Fluor594 (1:1000, Jackson #115-585-205), goat anti-rabbit IgG-Alexa488 (1:1000, Thermo#A11070), DAPI (Sigma #10236276001), α-Bungarotoxin (BTX) Alexa Fluor594 conjugate (1:300, ThermoFisher #B13423) and α-Bungarotoxin Alexa Fluor555 conjugate (ThermoFisher #B35451). The Image J software was employed for measuring the size of cross-sectional area of Laminin-stained fibers using the wand tool. The mean cross-sectional area was calculated from 200 fibers per animal. To determine the fiber size distribution the total number of fibres for each size-category (size range [%]) was counted.

Whole-mount staining of the diaphragm followed published protocols. Diaphragm muscles were isolated at E15.5 and E18.5 and stained with α-Bungarotoxin conjugated with Alexa Fluor 555 or 594. To label the presynaptic area, anti-SV2 (1:200, synaptic vesicles, DSHB #SV2) and anti-neurofilament (1:500, BioLegend #837904) antibodies were used. Afterwards, samples were flat-mounted and embedded using Fluoromount W (Serva #21634.01). The width of AChR endplate zone in BTX-stained whole diaphragm muscles was measured using ImageJ 1.53k. The average cross-distance was calculated for 25 different areas in the middle region of the endplate zone per diaphragm. The AChR cluster size and number was quantified either from serial TA and paraspinal muscle transversal sections or from whole mount diaphragm confocal microscope Z-stack data (Leica SP8). For neonatal muscles, serial paraspinal spinal muscle sections were stained with BTX, the synaptic band was localized, and eight randomised images were taken within the synaptic band zone (technical replicates, Zen 2.1, Zeiss Z1). Image J software was employed to count and measure the number of AChR clusters per image. The relative size of individual clusters within the images was calculated (BTX area per muscle area in %). The mean cluster size of all technical replicates was calculated for each animal. To determine cluster size distribution, the total number of clusters of each size-category (0 - 0.005 %, 0.005 % - 0.010

%, > 0.010 % size range) was counted. Z-stacked confocal images obtained from BTX-stained whole mount diaphragms (endplate band region, four technical replicates) were used to quantify the average number and volume of AChR clusters. 3D rendering and quantification was done using IMARIS x64 9.6.0). For adult TA muscles >450 fibers were counted and the number of AChR signals per fiber determined.

Mass spectrometry (MS)

Triplicate samples of E18.5 mouse quadriceps where lyzed in 4% SDS, 100 mM Tris, pH 7.6 for whole skeletal muscle proteomics using manual maceration with plastic douncers. After estimating protein content using DC Protein Assay (Biorad, Feldkirchen, Germany), 100 µg protein of *miR-1/206/133* tKO and wild type samples where acetone precipitated and resuspended in 6 M urea/2 M thiourea, 10 mM HEPES, pH 8.0, followed by in solution digestion. In brief, protein was reduced using 10 mM dithiothreitol and alkylated using 55 mM iodoacetamide. Next, proteins were digested using Lys-C (1:50; Wako Chemicals GmbH, Neuss, Germany) at room temperature for 3 h, followed by 1:4 dilution of the denaturants using 100 mM TEAB and overnight trypsinization (1:50; Serva, Heidelberg, Germany) at room temperature. After determining the concentration of the resulting peptide mixture using the Pierce Fluorimetric Peptide Assay (ThermoFisher Scientific, Dreieich, Germany), equal amounts of peptides where subjected to stable isotope labeling using reductive dimethylation [1, 2] using a labeling schedule in which wild type control samples (WT) where labeled “light” and combined in an experimental set with “heavy” labeled *miR-1/206/133* tKO. In brief, peptides were acidified by TFA and differentially modified using formaldehyde-H2 and cyanoborohydride (“light”), or formaldehyde-13C-D2 and cyanoborodeuteride (“heavy”), respectively. Ammonia and formic acid were used to quench the labeling reaction. Labeling efficiency was confirmed to be >95% by LC/MS2 and differentially labeled samples mixed 1:1, followed by subsequent desalting using oligo R3 columns. For greater sequencing depth, samples were separated into eight fractions with the Pierce High pH Reversed-Phase Peptide Fractionation Kit (ThermoFisher Scientific, Dreieich, Germany), according to the manufacturer's protocol, followed by final desalting, concentration and storage on STAGE tips [3].

Triplicate sample sets of affinity purified CRK from wild type, as well as *miR-1/206/133* tKO E18.5 limb muscle samples were subjected to in gel digest to detect interaction partners of CRK [4]. In brief, gel lanes were cut into blocks and finely diced. Proteins were subsequently reduced (10 mM dithiothreitol) and alkylated (55 mM iodoacetamide), followed by overnight digestion using trypsin (Serva, Heidelberg, Germany). Peptides were gradually eluted from the gel by increasing concentrations of acetonitrile and STAGE tip purified as above.

Processed samples were analyzed using liquid chromatography/tandem-mass spectrome-try (LC/MS2) using in in house-packed column emitters (15 cm, 70 µm ID, ReproSil-Pur 120 C18-AQ, 1.9 µm, Dr.

Maisch GmbH, Ammerbuch-Entringen Germany) and a buffer system comprising 1% formic acid, 80% acetonitrile, and 1% formic acid. Relevant instrumentation parameters were extracted using MARMoSET [5] and are included in the supplementary information (Supplementary Data 1). Peptide/spectrum matching, as well as quantitation was performed using the MaxQuant suite of algorithms (proteome: v. 1.6.0.1; interactome: v. 1.6.6.0) [6, 7, 8]. against the canonical and isoforms UniProt mouse database (proteome: downloaded 2017/04/20, 97484 entries; interactome: downloaded 2019/08/19, 86161 entries; [9]) using parameters documented in the supplementary material (Supplementary Data 1). Downstream bioinformatics analysis was performed using a limma-based R pipeline [10] (https://github.com/bhagwataditya/autonomics)).

a Mendelian distribution of offspring at the time of weaning

Line	Genotype	N	% obseved	% expected
**sKO**	miR-1-1/133a-2$^{+/+}$ miR-1-2/133a-1$^{+/+}$ miR-206/133b$^{+/+}$	49	25	25
	miR-1-1/133a-2$^{+/+}$ miR-1-2/133a-1$^{+/+}$ miR-206/133b$^{+/-}$	98	50	50
	miR-1-1/133a-2$^{+/+}$ miR-1-2/133a-1$^{+/+}$ miR-206/133b$^{-/-}$	48	25	25
	**Total**	**195**	**100**	**100**
**dKO**	miR-1-1/133a-2$^{-/-}$ miR-1-2/133a-1$^{lox/lox}$ miR-206/133b$^{+/+}$ Pax7-ICNm Cre$^{+/+}$	35	26	25
	miR-1-1/133a-2$^{+/-}$ miR-1-2/133a-1$^{lox/lox}$ miR-206/133b$^{+/+}$ Pax7-ICNm Cre$^{+/-}$	39	29	25
	miR-1-1/133a-2$^{+/-}$ miR-1-2/133a-1$^{lox/lox}$ miR-206/133b$^{+/+}$ Pax7-ICNm Cre$^{+/+}$	26	19	25
	miR-1-1/133a-2$^{-/-}$ miR-1-2/133a-1$^{lox/lox}$ miR-206/133b$^{+/+}$ Pax7-ICNm Cre$^{+/-}$	35	26	25
	**Total**	**135**	**100**	**100**
**tKO**	miR-1-1/133a-2$^{-/-}$ miR-1-2/133a-1$^{lox/lox}$ miR-206/133b$^{+/-}$ Pax7-ICNm Cre$^{+/-}$	9	26	25
	miR-1-1/133a-2$^{-/-}$ miR-1-2/133a-1$^{lox/lox}$ miR-206/133b$^{-/-}$ Pax7-ICNm Cre$^{+/+}$	10	29	25
	miR-1-1/133a-2$^{-/-}$ miR-1-2/133a-1$^{lox/lox}$ miR-206/133b$^{+/-}$ Pax7-ICNm Cre$^{+/+}$	15	44	25
	miR-1-1/133a-2$^{-/-}$ miR-1-2/133a-1$^{lox/lox}$ miR-206/133b$^{-/-}$ Pax7-ICNm Cre$^{+/-}$	0	0	25
	**Total**	**34**	**100**	**100**

b Mendelian distribution of offspring at E18.5 in tKO

Line	Genotype	N	% obseved	% expected
**tKO**	miR-1-1/133a-2$^{-/-}$ miR-1-2/133a-1$^{lox/lox}$ miR-206/133b$^{+/-}$ Pax7-ICNm Cre$^{+/-}$	4	13	25
	miR-1-1/133a-2$^{-/-}$ miR-1-2/133a-1$^{lox/lox}$ miR-206/133b$^{-/-}$ Pax7-ICNm Cre$^{+/+}$	10	31	25
	miR-1-1/133a-2$^{-/-}$ miR-1-2/133a-1$^{lox/lox}$ miR-206/133b$^{+/-}$ Pax7-ICNm Cre$^{+/+}$	12	38	25
	miR-1-1/133a-2$^{-/-}$ miR-1-2/133a-1$^{lox/lox}$ miR-206/133b$^{-/-}$ Pax7-ICNm Cre$^{+/-}$	6	19	25
	**Total**	**32**	**100**	**100**

Supplementary table 1: Skeletal muscle specific deletion of *miR-1/206/133* miRNAs causes neonatal lethality.

a-b Numbers of *miR-206/133b* sKO and skeletal muscle specific *miR-1/133a* knock-out animals (dKO) at postnatal day 21 (P21) in relation to expected Mendelian ratios. No surviving skeletal muscle specific *miR-1/206/133* deficient tKO animals were detected at P21 (red). In contrast, the numbers of skeletal muscle specific *miR-1/206/133* tKO animals match the expected ratios at embryonic day 18.5 (E18.5) (P21: n = 147/48 for control littermates (sKO), n = 100/35 for control littermates (dKO), n = 34/0 for control littermates (tKO); E18.5: n = 26/6 for control littermates (tKO); numbers of *miR-1/206/133* tKO animals are in blue).

a

miRNA	target gene	target gene ID	p value	FC	miRNA start	miRNA end	gene start	gene end	align score	energy
**mmu-miR-1**	**Crk**	12928	0.00	1.4	2	19.0	749.0	770.0	126.0	-14.8
**mmu-miR-1**	**Crk**	12928	0.00	1.4	2	19.0	619.0	640.0	126.0	-14.8
**mmu-miR-133**	**Crk**	12928	0.00	1.4	2	20.0	863.0	888.0	149.0	-18.7
**mmu-miR-133**	**Crk**	12928	0.00	1.4	2	20.0	1250.0	1270.0	141.0	-22.5
**mmu-miR-206**	**Crk**	12928	0.00	1.4	2	21.0	751.0	770.0	121.0	-14.2
**mmu-miR-206**	**Crk**	12928	0.00	1.4	2	21.0	621.0	640.0	121.0	-14.2
mmu-miR-1	Dcx	13193	0.00	5.9	2	21.0	5347.0	5369.0	135.0	-15.9
mmu-miR-133	Dcx	13193	0.00	5.9	2	20.0	294.0	315.0	125.0	-17.0
mmu-miR-133	Dcx	13193	0.00	5.9	2	15.0	4097.0	4118.0	122.0	-16.9
mmu-miR-133	Dcx	13193	0.00	5.9	2	18.0	1154.0	1175.0	121.0	-20.6
mmu-miR-206	Dcx	13193	0.00	5.9	2	17.0	5348.0	5369.0	128.0	-15.5
mmu-miR-1	Mtpn	14489	0.00	1.5	2	21.0	2026.0	2048.0	139.0	-18.7
mmu-miR-133	Mtpn	14489	0.00	1.5	2	8.0	505.0	526.0	120.0	-17.2
mmu-miR-206	Mtpn	14489	0.00	1.5	2	21.0	2026.0	2048.0	136.0	-20.6
mmu-miR-206	Mtpn	14489	0.00	1.5	2	20.0	3024.0	3049.0	122.0	-16.4
mmu-miR-1	Gdi1	14567	0.00	1.6	2	9.0	107.0	128.0	140.0	-14.4
mmu-miR-133	Gdi1	14567	0.00	1.6	2	18.0	761.0	785.0	120.0	-16.3
mmu-miR-206	Gdi1	14567	0.00	1.6	2	18.0	109.0	128.0	146.0	-17.2
mmu-miR-206	Gdi1	14567	0.00	1.6	2	19.0	505.0	526.0	138.0	-20.4
mmu-miR-1	Gpd2	14571	0.02	1.5	2	21.0	287.0	309.0	143.0	-14.9
mmu-miR-133	Gpd2	14571	0.02	1.5	2	18.0	1337.0	1360.0	159.0	-28.1
mmu-miR-133	Gpd2	14571	0.02	1.5	2	19.0	2633.0	2657.0	128.0	-17.0
mmu-miR-206	Gpd2	14571	0.02	1.5	2	21.0	287.0	309.0	151.0	-19.2
mmu-miR-206	Gpd2	14571	0.02	1.5	2	21.0	68.0	89.0	136.0	-14.4
mmu-miR-1	Nras	18176	0.00	1.5	2	21.0	2098.0	2118.0	142.0	-15.5
mmu-miR-133	Nras	18176	0.00	1.5	2	8.0	1192.0	1213.0	120.0	-19.3
mmu-miR-206	Nras	18176	0.00	1.5	2	9.0	1951.0	1972.0	140.0	-14.9
mmu-miR-206	Nras	18176	0.00	1.5	2	20.0	2096.0	2118.0	138.0	-18.6
mmu-miR-1	Dlg2	23859	0.00	1.4	2	21.0	337.0	365.0	122.0	-16.3
mmu-miR-133	Dlg2	23859	0.00	1.4	2	18.0	1417.0	1439.0	144.0	-17.2
mmu-miR-206	Dlg2	23859	0.00	1.4	2	21.0	4412.0	4434.0	127.0	-19.9
mmu-miR-1	Sacs	50720	0.00	1.4	2	21.0	81.0	104.0	175.0	-20.2
mmu-miR-1	Sacs	50720	0.00	1.4	2	21.0	1219.0	1239.0	146.0	-16.4
mmu-miR-133	Sacs	50720	0.00	1.4	2	21.0	881.0	906.0	122.0	-19.5
mmu-miR-206	Sacs	50720	0.00	1.4	2	21.0	83.0	104.0	168.0	-21.0
mmu-miR-206	Sacs	50720	0.00	1.4	2	21.0	1219.0	1239.0	138.0	-16.3
mmu-miR-1	Slc1a4	55963	0.00	1.8	2	20.0	1184.0	1205.0	156.0	-16.4
mmu-miR-133	Slc1a4	55963	0.00	1.8	2	11.0	266.0	287.0	150.0	-24.4
mmu-miR-206	Slc1a4	55963	0.00	1.8	2	21.0	1182.0	1205.0	154.0	-19.6
mmu-miR-1	Slc25a23	66972	0.01	1.7	2	18.0	732.0	757.0	151.0	-15.7
mmu-miR-133	Slc25a23	66972	0.01	1.7	2	17.0	1412.0	1436.0	142.0	-15.5
mmu-miR-206	Slc25a23	66972	0.01	1.7	2	10.0	736.0	757.0	145.0	-17.1
mmu-miR-206	Slc25a23	66972	0.01	1.7	2	17.0	1529.0	1551.0	143.0	-14.7
mmu-miR-206	Slc25a23	66972	0.01	1.7	2	20.0	1237.0	1260.0	154.0	-19.2
mmu-miR-1	Igf2bp3	140488	0.00	1.6	2	18.0	393.0	415.0	124.0	-14.3
mmu-miR-133	Igf2bp3	140488	0.00	1.6	2	21.0	847.0	871.0	127.0	-19.3
mmu-miR-206	Igf2bp3	140488	0.00	1.6	2	20.0	395.0	415.0	129.0	-15.0
mmu-miR-1	Lpp	210126	0.00	1.4	2	20.0	270.0	291.0	143.0	-15.9
mmu-miR-133	Lpp	210126	0.00	1.4	2	19.0	1180.0	1202.0	125.0	-14.6
mmu-miR-133	Lpp	210126	0.00	1.4	2	8.0	1460.0	1481.0	120.0	-15.1
mmu-miR-206	Lpp	210126	0.00	1.4	2	21.0	272.0	291.0	149.0	-22.4
mmu-miR-1	Dzip3	224170	0.00	1.4	2	13.0	2474.0	2495.0	156.0	-14.4
mmu-miR-133	Dzip3	224170	0.00	1.4	2	8.0	1338.0	1359.0	120.0	-14.5
mmu-miR-206	Dzip3	224170	0.00	1.4	2	13.0	2474.0	2495.0	152.0	-14.1
mmu-miR-1	Raver2	242570	0.00	1.4	2	21.0	1686.0	1706.0	154.0	-15.4
mmu-miR-133	Raver2	242570	0.00	1.4	2	10.0	631.0	652.0	145.0	-21.6
mmu-miR-206	Raver2	242570	0.00	1.4	2	21.0	1688.0	1706.0	160.0	-20.7
mmu-miR-206	Raver2	242570	0.00	1.4	2	9.0	231.0	252.0	124.0	-17.4

Supplementary table 2: Predicted *miR-1/206/133* target genes upregulated in *miR-1/206/133* tKO

a List of significantly up-regulated *miR-1/206/133* target genes in *miR-1/206/133* tKO muscles. The NMJ-associated *Crk* gene (blue) was selected as top candidate for further investigations. Criteria for selection were: (i) upregulated in tKO with a log_2 fold change (FC) of >1.35 and a p-value of <0.05 (ii) predicted target gene of *miR-1*, *miR-206* and *miR-133* (microRNA:target interaction energy <-14).

a Mendelian distribution of offspring at the time of weaning

Line	Genotype	N	% obseved	% expected
**Crk-SPH**	$sgRNA^{+/-}$ $dCAS9^{+/-}$ Pax7-ICNm $Cre^{+/+}$	3	7.9	12.5
	$sgRNA^{+/-}$ $dCAS9^{+/+}$ Pax7-ICNm $Cre^{+/-}$	7	18.4	12.5
	$sgRNA^{+/+}$ $dCAS9^{+/-}$ Pax7-ICNm $Cre^{+/+}$	8	21.1	12.5
	$sgRNA^{+/+}$ $dCAS9^{+/-}$ Pax7-ICNm $Cre^{+/-}$	4	10.5	12.5
	$sgRNA^{+/+}$ $dCAS9^{+/+}$ Pax7-ICNm $Cre^{+/-}$	4	10.5	12.5
	$sgRNA^{+/+}$ $dCAS9^{+/+}$ Pax7-ICNm $Cre^{+/+}$	4	10.5	12.5
	$sgRNA^{+/-}$ $dCAS9^{+/+}$ Pax7-ICNm $Cre^{+/+}$	8	21.1	12.5
	$sgRNA^{+/-}$ $dCAS9^{+/-}$ Pax7-ICNm $Cre^{+/-}$	0	0	12.5
	**Total**	**38**	**100**	**100**

Supplementary table 3: Skeletal muscle specific overexpression of *Crk* causes neonatal lethality.

a Numbers of *Crk-SPH* animals at postnatal day 21 (P21) in relation to expected Mendelian ratios. No surviving *Crk-SPH* animals (green; $sgRNA^{+/-}//dCas9^{+/-}//Pax7\text{-}Cre^{+/-}$) were detected at the time of weaning (P21), (n = 38/0; control = littermates).

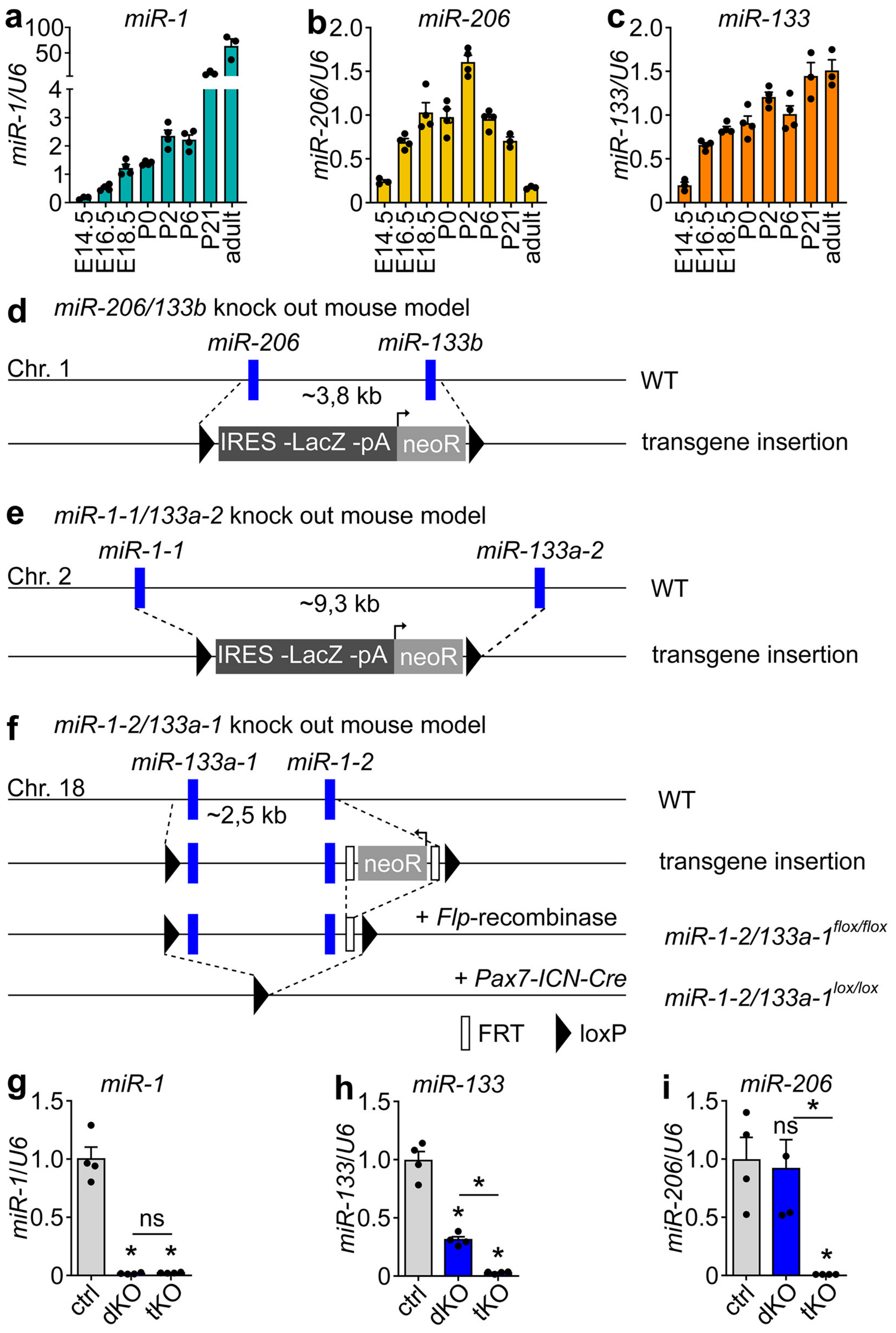

Supplementary figure 1: Strategy to generate the *miR-1/206/133* knockout mouse model.

a-c Developmental profile of *miR-1*, *miR-206*, and *miR-133* expression in wild-type quadriceps muscles analyzed by TaqMan assays (E14.5, n = 3 [for each biological replicate the quadriceps muscles of 3 animals were pooled], E16.5-P6 n = 4, P21-adult n=3 (male), Mann-Whitney U test, one-tailed, Data are presented as mean values +/- SEM). *U6 snRNA* served as control.

d-f Schematic illustration of the genomic loci of *miR-206/133b*, *miR-1-1/133a-2* and *miR-1-2/133a-1*. The *miR-206/133b* and the *miR-1-1/133a-2* clusters were deleted by insertion of a PGK-neomycin selection cassette. *miR-1-1/133a-2* mutant mice were mated to *Pax7-ICN-Cre* mice to generate muscle specific *miR-1/133a* mutants.

g-i Analysis of *miR-1*, *miR-133*, and *miR-206* expression using TaqMan assays in *miR-1/206/133* tKO, *miR-1/133a* dKO and control quadriceps muscles at E18.5 (*miR-1/133* TaqMan assay n = 4 control/4 dKO/4 tKO, *miR-206* TaqMan assay n = 4 control/3 dKO/4 tKO, Kolmogorov–Smirnov U test, two-tailed, *p <0.0286, ns = not significant, Data are presented as mean values +/- SEM, control: *miR-1-1/133a-2*$^{+/+}$, *miR-1-2/133a-1*$^{lox/lox}$, *miR-206/133b*$^{+/+}$, *Pax7-Cre*$^{+/+}$). *U6 snRNA* served as control. Source data are provided as a Source Data file.

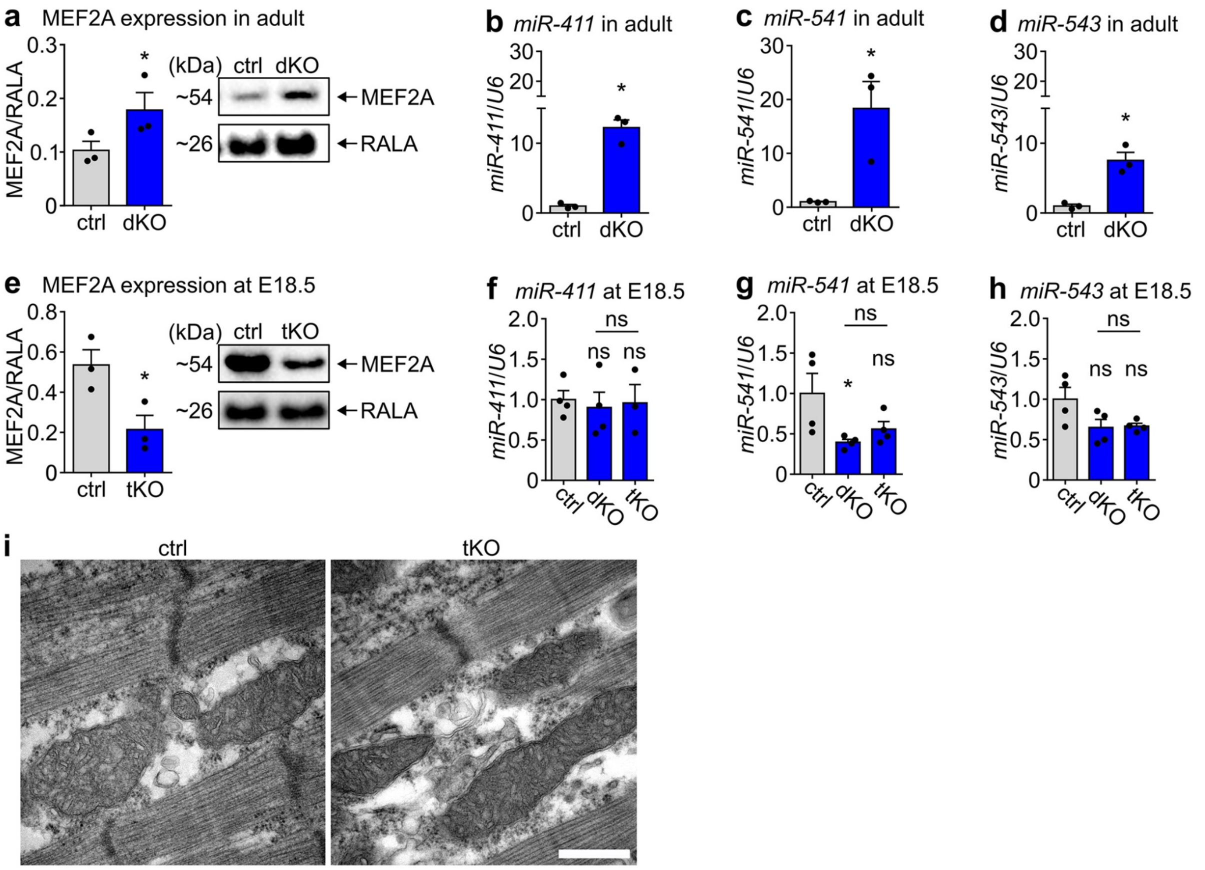

Supplementary figure 2: Skeletal muscle specific deletion of *miR-1/133a* or *miR-1/206/133* affects the MEF2A - *Dlk1-Dio3* axis in adults but not at embryonic day 18.5.

a Western blot analysis shows increased MEF2A expression in adult *miR-1/133a* dKO (n = 3) muscles, corroborating previous data (n = 3, control: *miR-1-1/133a-2*$^{+/+}$*//miR-1-2/133a-1*$^{lox/lox}$*//miR-206/133b*$^{+/+}$*// Pax7-Cre*$^{+/+}$, Mann-Whitney U test, one-tailed, *p = 0.05, Data are presented as mean values +/- SEM, quadriceps muscles [14-18 weeks, males and females]).

b-d TaqMan assay demonstrating upregulation of individual miRNAs (*miR-411*, *miR-541*, *miR-543*) of the *Dlk1-Dio3* mega-cluster in adult *miR-1/133a* dKO (n = 3) compared to control (n = 3) as demonstrated previously (control: *miR-1-1/133a-2*$^{+/+}$*//miR-1-2/133a-1*$^{lox/lox}$*//miR-206/133b*$^{+/+}$*//Pax7-Cre*$^{+/+}$, Mann-Whitney U test, one-tailed, *p = 0.05, Data are presented as mean values +/- SEM, quadriceps muscles [14-18 weeks, males and females]).

e Western blot analysis showing MEF2A expression in *miR-1/206/133* tKO (n = 3) at E18.5 (n = 3, control: *miR-1-1/133a-2*$^{+/+}$*//miR-1-2/133a-1*$^{+/+}$*//miR-206/133b*$^{+/+}$*//Pax7-Cre*$^{+/+}$, Mann-Whitney U test, one-tailed, *p = 0.05, Data are presented as mean values +/- SEM, quadriceps muscles [E18.5]).

f-h TaqMan assay showing normal expression of individual miRNA genes (*miR-411*, *miR-541*, *miR-543*) within the *Dlk1-Dio3* mega-cluster in *miR-1/133a* dKO (n = 4) or *miR-1/206/133* tKO (n = 4) compared to control (n = 3) quadriceps muscles at E18.5 (control: *miR-1-1/133a-2*$^{+/+}$*//miR-1-2/133a-1*$^{lox/lox}$*//miR-206/133b*$^{+/+}$*//Pax7-Cre*$^{+/+}$, Mann-Whitney U test, one-tailed, *p = 0.0143, Data are presented as mean values +/- SEM, ns = not significant).

i Histological examination of *miR-1/206/133* tKO (n = 3) and control (n = 3) quadriceps muscles. Electron microscopy reveals no change in the mitochondrial ultrastructure in longitudinal sections (scale bar: 1000 nm [E18.5]). Source data are provided as a Source Data file.

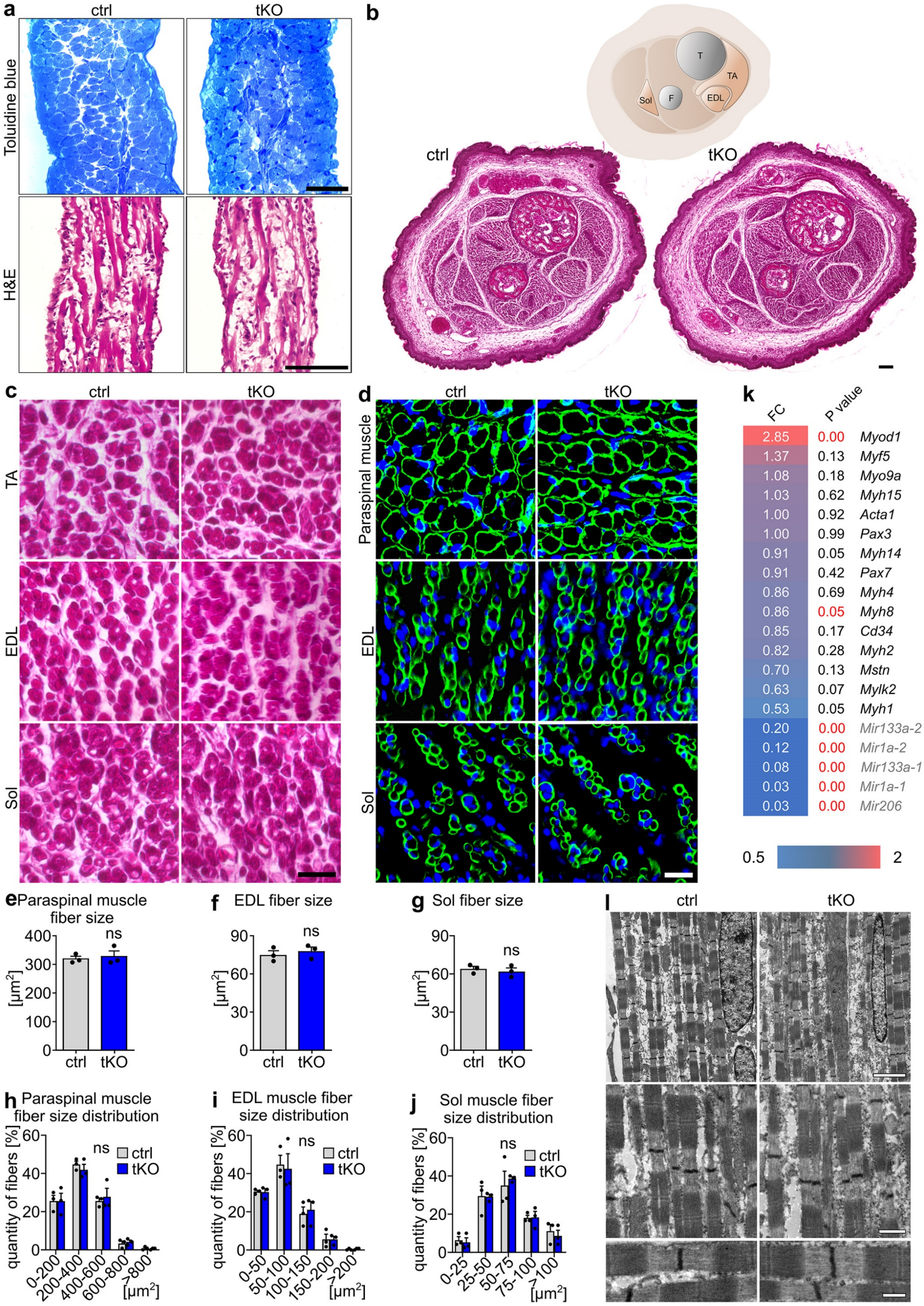
a
ctrl
tKO
Toluidine blue
H&E
b
T
TA
Sol
F
EDL
ctrl
tKO
c
ctrl
tKO
TA
EDL
Sol
d
ctrl
tKO
Paraspinal muscle
EDL
Sol
k
FC
P value
2.85 0.00 Myod1
1.37 0.13 Myf5
1.08 0.18 Myo9a
1.03 0.62 Myh15
1.00 0.92 Acta1
1.00 0.99 Pax3
0.91 0.05 Myh14
0.91 0.42 Pax7
0.86 0.69 Myh4
0.86 0.05 Myh8
0.85 0.17 Cd34
0.82 0.28 Myh2
0.70 0.13 Mstn
0.63 0.07 Mylk2
0.53 0.05 Myh1
0.20 0.00 Mir133a-2
0.12 0.00 Mir1a-2
0.08 0.00 Mir133a-1
0.03 0.00 Mir1a-1
0.03 0.00 Mir206
0.5
2
e Paraspinal muscle fiber size
f EDL fiber size
g Sol fiber size
[µm²]
ns
ctrl tKO
h Paraspinal muscle fiber size distribution
i EDL muscle fiber size distribution
j Sol muscle fiber size distribution
quantity of fibers [%]
ctrl
tKO
ns
0-200 200-400 400-600 600-800 >800 [µm²]
0-50 50-100 100-150 150-200 >200 [µm²]
0-25 25-50 50-75 75-100 >100 [µm²]
l
ctrl
tKO

Supplementary figure 3: Neonatal *miR-1/206/133* tKO mice show normal skeletal muscle development

a Histological examination of *miR-1/206/133* tKO (n = 2, right) and control (n = 2, littermates, left,) diaphragms. Semi thin longitudinal sections stained with toluidine blue (upper row, scale bar: 10 µm). H&E staining on transversal sections (lower row, scale bar: 50 µm).

b-c H&E staining of control (n = 3) and tKO (n = 3) lower limb muscles, transversal paraffin sections. Scheme indicates position of T (*tibia*), F (*femur*), TA (m. *tibialis anterior*), EDL (m. *extensor digitorum longus*), Sol (m. *soleus*) within the lower leg. H&E staining of control (littermates) and *miR-1/206/133* tKO TA, EDL, Sol muscles using transversal sections (**c**). Scale bar: 100 µm (**b**) / 20 µm (**c**).

d-j Laminin (green) and DAPI (blue) immunostaining of transversal sections from paraspinal, EDL, Sol muscles of control (n = 3) and *miR-1/206/133* tKO (n = 3) mice (control= littermate, scale bar: 20 µm) (**d**). Average fiber size (cross sectional area, **e-g**) and fiber size distribution (**h-j**) (ns = not significant, Mann-Whitney U test, one-tailed, Data are presented as mean values +/- SEM).

k Transcriptome data of genes involved in myogenic development and differentiation in control (n = 4) and *miR-1/206/133* tKO (n = 4) skeletal muscles (Affymetrix microarray analysis, control=littermate, FC = $\log_2$ fold change, modified t-test, red = significant).

l Electron microscopy of longitudinal sections from *miR-1/206/133* tKO (n = 3) and control (n = 3) quadriceps muscles (control = WT, scale bar: upper row 5200 nm, middle row 1000 nm, lower row 500 nm). Source data are provided as a Source Data file.

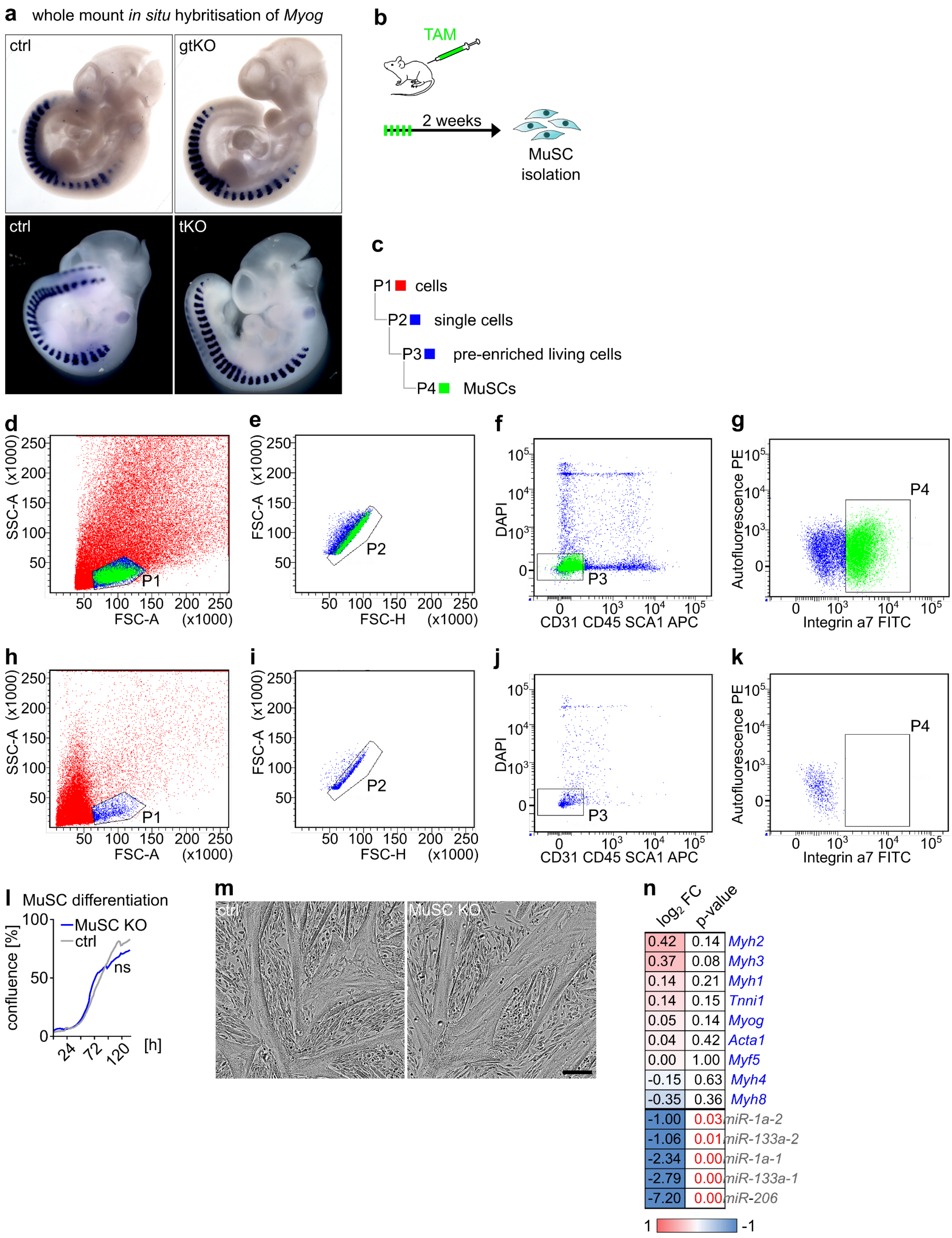

Supplementary figure 4: Normal embryonic skeletal muscle development and ex vivo differentiation of MuSC in absence of miR-1/206/133.

a Whole mount in situ (WISH) expression analysis of *Myog* in conditional *miR-1/206/133* tKO and constitutive *miR-1/206/133* germline knock-out (gtKO) embryos and littermates at E10.5.

b-k Isolation of tamoxifen (TAM) induced *miR-1/206/133* knock-out in muscle stem cells (MuSC tKO) using FACS. Green rectangles indicate injection days (**b**). Gating strategy identifying cells, singlets, using pre-enriched and live cells to detect MuSCs positive for Integrin a7 – FITC (**c-k**). MuSCs population was

defined using FMO (Fluorescense Minus One) FITC control (**h-k**). CD31-APC, CD45-APC and SCA1-APC population were depleted using magnetic columns on AutoMACS (Miltenyi), and dead cells were excluded using DAPI (**f,j**). Pre-enriched fraction was used to sort Integrin-a7 FITC positive MuSC population (9.6% of all detected cells) (**g**). APC = allophycocyanin; FITC = fluorescein isothiocyanate; PE = phycoerythrin.

l-n *miR-1/206/206*-deficient muscle stem cells (MuSC tKO, n = 3) differentiate normally to myotubes and show no difference in cell confluence after 120 h in culture (control = 2, scale-bar: 150 µm) (**l-m**). *miR-1/206/133* tKO MuSC show no changes in the expression of selected myogenic factors (**n**) Affymetrix microarray analysis, $\log_2$ FC = $\log_2$ fold change, modified t-test, red = significant, control = *miR1-1/133a-2*$^{-/-}$ *//miR-1-2/133a-1*$^{lox/lox}$*//miR-206/133b*$^{-/-}$ *//Pax7-CreERT2*$^{+/+}$ [10-16 weeks; male, female]. Source data are provided as a Source Data file.

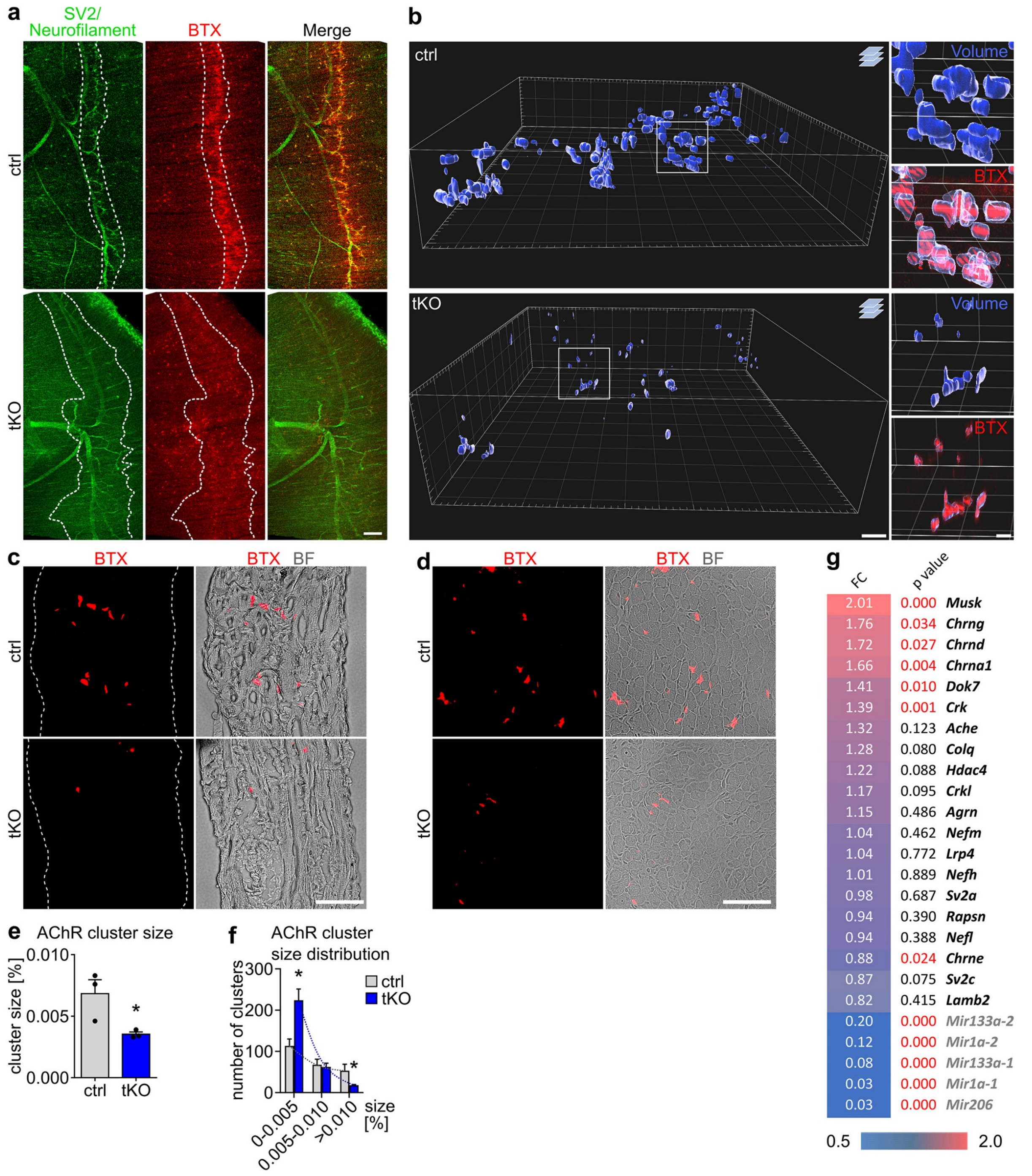

Supplementary figure 5: Skeletal muscle-specific loss of *miR-1/206/133* disturbs pre-and postsynaptic neuromuscular synapse formation.

a Whole-mount staining of hemi-diaphragms from control (n = 1, littermate) and *miR-1/206/133* tKO mice (n = 2). Nerve terminals and axons (green) were labeled with anti-SV2 (synaptic vesicles) and anti-neurofilament antibodies, respectively. Postsynapses (red) were visualized by BTX staining (scale bar: 100 µm [E15.5]).

b 3D rendered confocal Z-stacks of BTX-stained whole mount diaphragms from *miR-1/206/133* tKO (n = 3) and control (n = 3, littermates). Volume (blue) of BTX (red) stained AChR clusters in Z-stacks, scale-bar: 20 µm/ 5 µm.

c-d Fluorescence and phase contrast images of BTX-stained postsynaptic AChR clusters in control (upper row) and *miR-1/206/133* tKO (lower row) transversal sections of diaphragm (n =1 control/1

tKO) and paraspinal muscles (n = 3 control/3 tKO), (control = *miR-1-1/133a-2*$^{+/+}$*//miR-1-2/133a-1*$^{lox/lox}$*//miR-206/133b*$^{+/+}$*//Pax7-Cre*$^{+/+}$, scale bar: 50 µm [E18.5]), BF = bright-field.

e Quantification of mean AChR cluster sizes in transversal sections of paraspinal muscles from control (n = 3) and *miR-1/206/133* tKO (n = 3) mice (control = *miR-1-1/133a-2*$^{+/+}$*//miR-1-2/133a-1*$^{lox/lox}$*//miR-206/133b*$^{+/+}$*//Pax7-Cre*$^{+/+}$, Mann-Whitney U test, one-tailed, *p = 0.05, Data are presented as mean values +/- SEM [E18.5])

f Distribution of AChR cluster sizes in transversal sections of paraspinal muscles from control (n = 3) and *miR-1/206/133* tKO (n = 3) mice (control = *miR-1-1/133a-2*$^{+/+}$*//miR-1-2/133a-1*$^{lox/lox}$*//miR-206/133b*$^{+/+}$*//Pax7-Cre*$^{+/+}$, Mann-Whitney U test, one-tailed, *p = 0.05, Data are presented as mean values +/- SEM [E18.5]).

g Transcriptome data of synaptic marker genes in control (n = 4) and *miR-1/206/133* tKO (n = 4) skeletal muscles (Affymetrix microarray analysis, control=littermate, control, FC = log_2 fold change, modified t-test, red = significant). Source data are provided as a Source Data file.

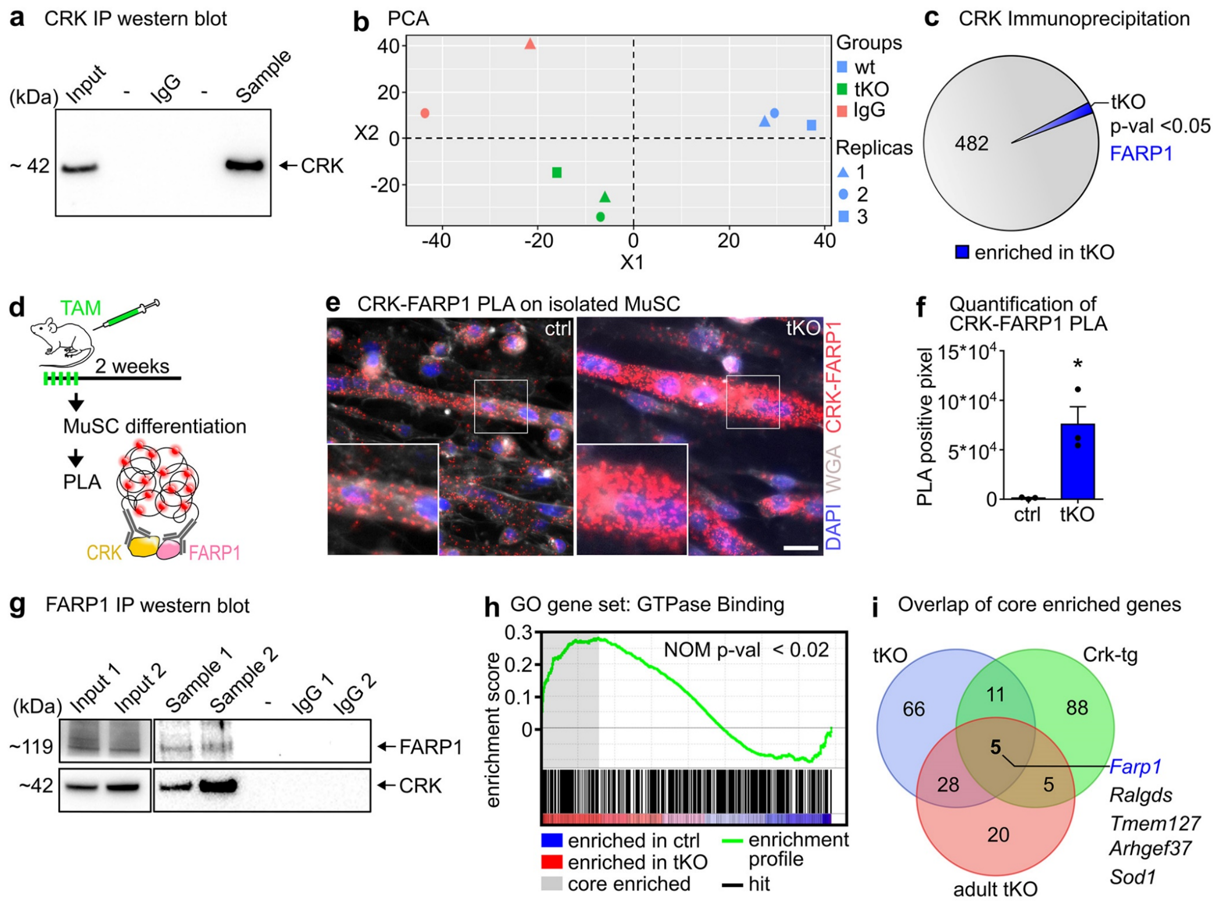

Supplementary figure 6: Increased interaction of CRK with RAC1-GEF FARP1 in *miR-1/206/133* tKO muscles.

a Immunoprecipitation (IP) of CRK using a CRK specific antibody. The input corresponds to 10% of protein lysate from P0 WT limb muscle tissue (n = 1).

b-c IP of CRK followed by MS analysis reveals significant enrichment of RAC1-GEF FARP1 protein in tKO mice (limb muscle tissue of WT n = 3 and tKO mice n = 3, [E18.5]). The CRK-IP MS analysis detected 492 potential interaction partners of CRK in WT and tKO. Ten proteins were significantly enriched in tKO samples. FARP1 was the only GEF in tKO muscles with increased CRK-interaction. Principal component analysis (PCA) demonstrates clustering of all replicates and separation of sample groups.

d-f Proximity ligation assays (PLA) detecting close proximity of two proteins *in situ* (**d**). **e-f** PLA confirms proximity of endogenous CRK to the RAC1-GEF FARP1 in the cytoplasm of differentiated MuSCs and reveals significant increase of PLA signals in differentiated *miR-1/206/133* tKO (n = 3) muscle stem cells (MuSCs) (n = 3 control, Mann-Whitney U test, one-tailed, *p = 0.05, Data are presented as mean values +/- SEM, independent MuSC isolations, two technical replicates for each animal, control = *miR-1-1/133a-2*$^{-/-}$*//miR-1-2/133a-1*$^{lox/lox}$*//miR-206/133b*$^{-/-}$*//Pax7-CE Cre*$^{+/+}$,MuSC tKO = *miR-1-1/133a-2*$^{-/-}$ *//miR-1-2/133a-1*$^{lox/lox}$*//miR-206/133b*$^{-/-}$*//Pax7-CE Cre*$^{+/-}$ scale = 20 µm [male 10 -14 weeks]).

g Co-IP of CRK and FARP1 using a FARP1 specific antibody. The input corresponds to 10% of protein lysate from P0 WT limb muscle tissue. CRK was detected by a CRK specific antibody (n = 2).

h GSEA of control and *miR-1/206/133* tKO (n = 4) quadriceps muscle transcriptome data reveals enrichment of transcripts involved in 'GTPase binding' (n = 4, control = *miR-1-1/133a-2*$^{+/+}$*//miR-1-2/133a-1*$^{lox/lox}$*//miR-206/133b*$^{+/+}$*//Pax7-Cre*$^{+/+}$, nominal p value (NOM p-val), p = 0.018 [E18.5]).

i Overlap of core enriched genes from the GSEA gene set 'GTPase Binding' in neonatal *miR-1/206/133* tKO, adult *miR-1/206/133* tKO, and neonatal *Crk-tg* animals. The RAC1-GEF *Farp1* is among the five genes enriched in all three models. Core enriched genes used in Venn diagram: GSEA of *miR-1/206/133* tKO (n = 4), *Crk-tg* (n = 3) and adult *miR-1/206/133* tKO (n = 3) transcriptome data (tKO: control vs. *miR-1/206/133* tKO quadriceps muscle [n = 4, control = *miR-1-1/133a-2*$^{+/+}$*//miR-1-2/133a-1*$^{lox/lox}$*//miR-206/133b*$^{+/+}$*//Pax7-Cre*$^{+/+}$, E18.5]/ *Crk-tg*: control vs. *Crk-tg* quadriceps muscles [n = 3, WT = littermates, E18.5]/ adult tKO: *miR-1/206/133* adult tKO vs. control TA muscle [n = 2, control= *miR-1-1/133a-2*$^{-/-}$ *//miR-1-2/133a-1*$^{lox/lox}$*//miR-206/133b*$^{-/-}$*//HSA-rtTA-TRE-Cre*$^{+/+}$, male, ~44 weeks]). Source data are provided as a Source Data file.

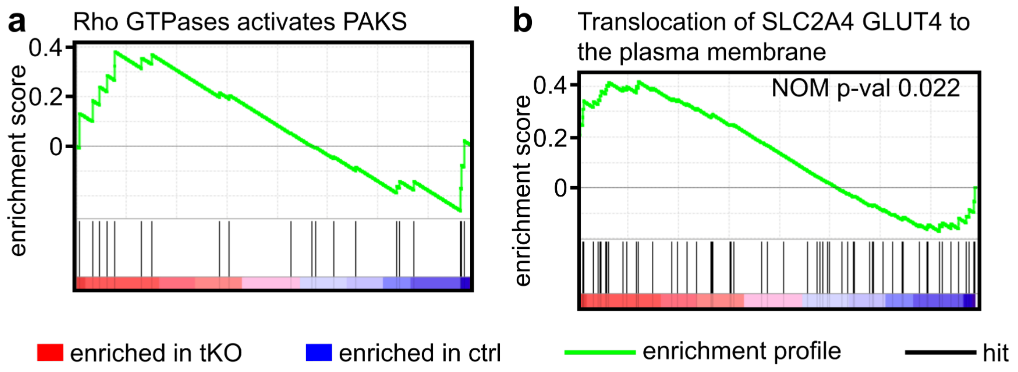

Supplementary figure 7: Activation of RAC-GTP downstream targets in neonatal *miR-1/206/133* tKO muscles

a-b GSEA using transcriptome data of neonatal *miR-1/206/133* tKO (n = 4) vs. control quadriceps muscles (n = 4, control = *miR-1-1/133a-2*$^{+/+}$//*miR-1-2/133a-1*$^{lox/lox}$//*miR-206/133b*$^{+/+}$//*Pax7-Cre*$^{+/+}$, nominal p value (NOM p-val), p = 0.022 [E18.5]).

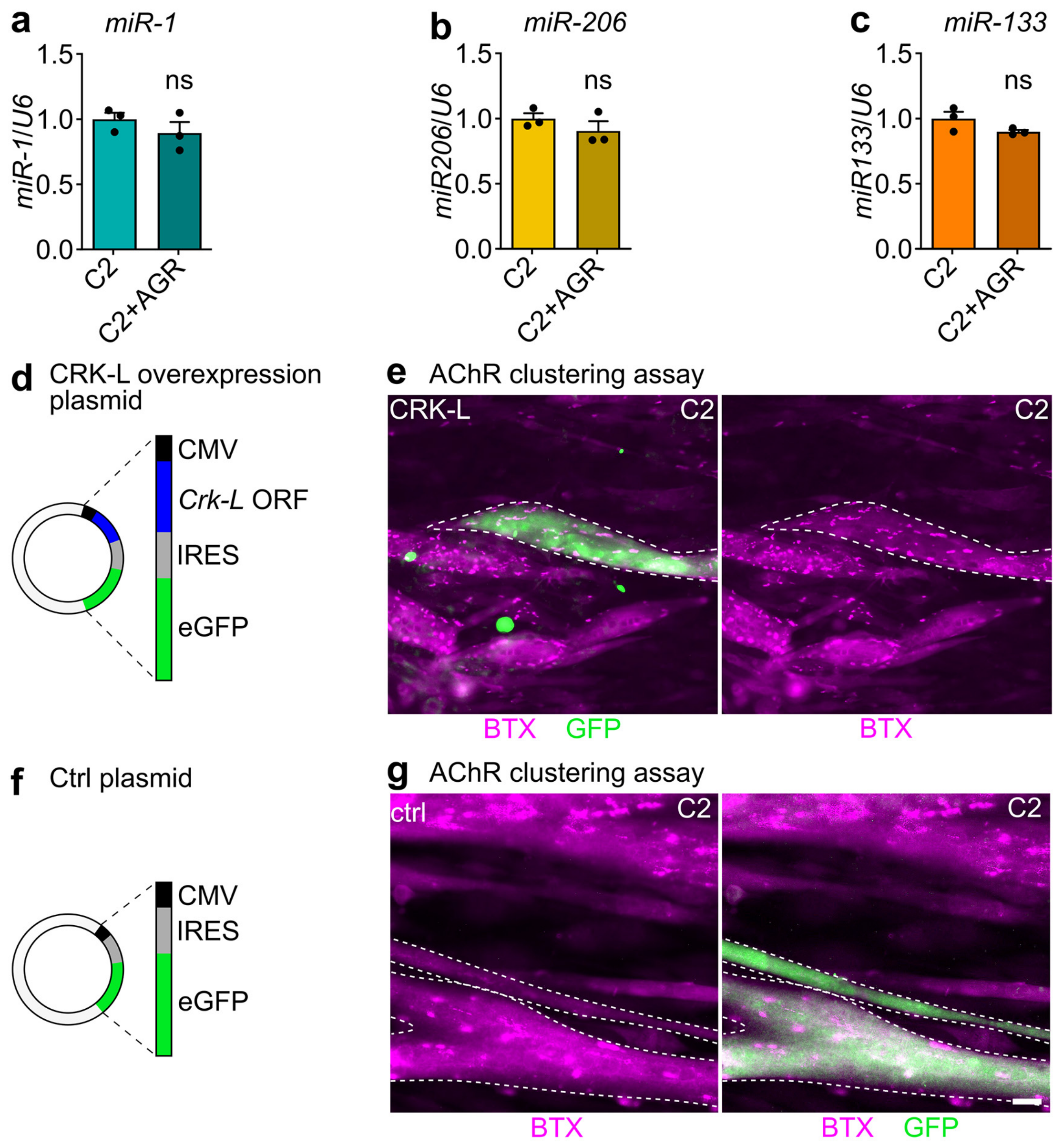

Supplementary figure 8: Increased levels of CRK-L do not impair AChR clustering.

a-c Endogenous *miR-1* (n = 3 control /3 C2+AGR), *miR-206* (n = 3 control /3 C2+AGR) and *miR-133* (n = 3 control/3 C2+AGR) expression in C2 cells after 16 h of stimulation by Agrin (AGR). (control = unstimulated C2 cells, TaqMan assay, biological replicates, ns = not significant, Mann-Whitney-U test, one-tailed, Data are presented as mean values +/- SEM). *U6 snRNA* served as control.

d-g Overexpression of *Crk-l* in C2 myotubes using a vector, also expressing GFP (**d**). The vector used in (d), but without *Crk-l* insert, served as control (**f**). Transfected myotubes were incubated with Agrin to induce AChR clustering (stained by Alexa Fluor594-conjugated BTX). GFP-positive (anti-GFP-Alexa488) myotubes show the same degree of AChR clustering as observed in non-transfected fibers (**e**,**g**, scale bar: 20 µm). Dashed lines mark myotubes expressing *Crk-l-GFP* (**d**) and *GFP* (**g**). Minimum n = 6 (biological replicate= independent transfection). Source data are provided as a Source Data file.

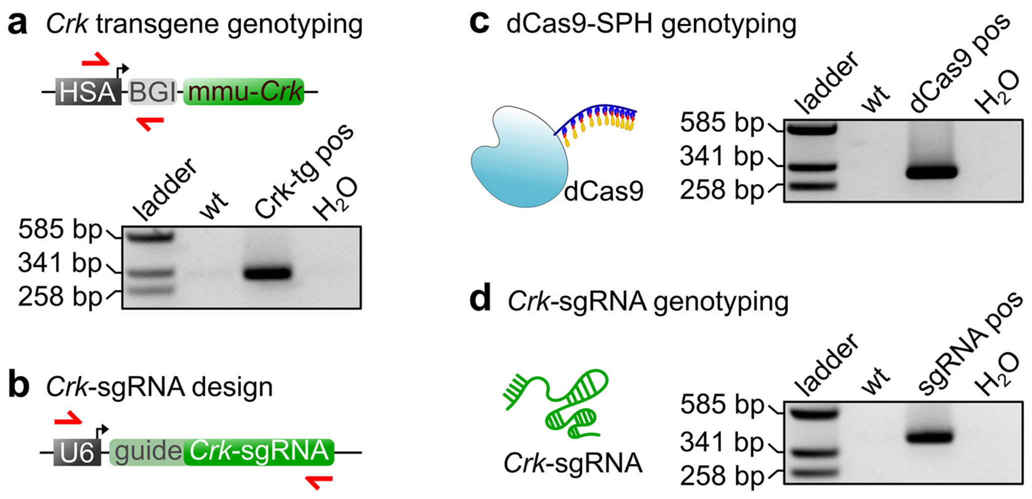

Supplementary figure 9: Genotyping of *Crk*-overexpressing mice.

a Localization of oligonucleotides used for genotyping of *Crk*-overexpressing transgenic mice (*Crk-tg*). Muscle specific overexpression of *Crk* is achieved via the human α-skeletal actin (HSA) promoter (BGI = intron of β-globin gene, mmu-*Crk* = ORF of *Crk*). The size of the amplified PCR-Product is 314 base pairs (bp). Positions of forward and reverse primers are indicated by red arrows.

b Design of *Crk* specific sgRNA (U6 = U6 promoter). Positions of forward and reverse primers are indicated by red arrows.

c-d Genotyping of *Crk*-sgRNA and dCas9-SPH positive animals. The size of the PCR product for the sgRNA is ~400 bp and 305 bp (bp = base pairs) for dCas9. Source data are provided as a Source Data file.

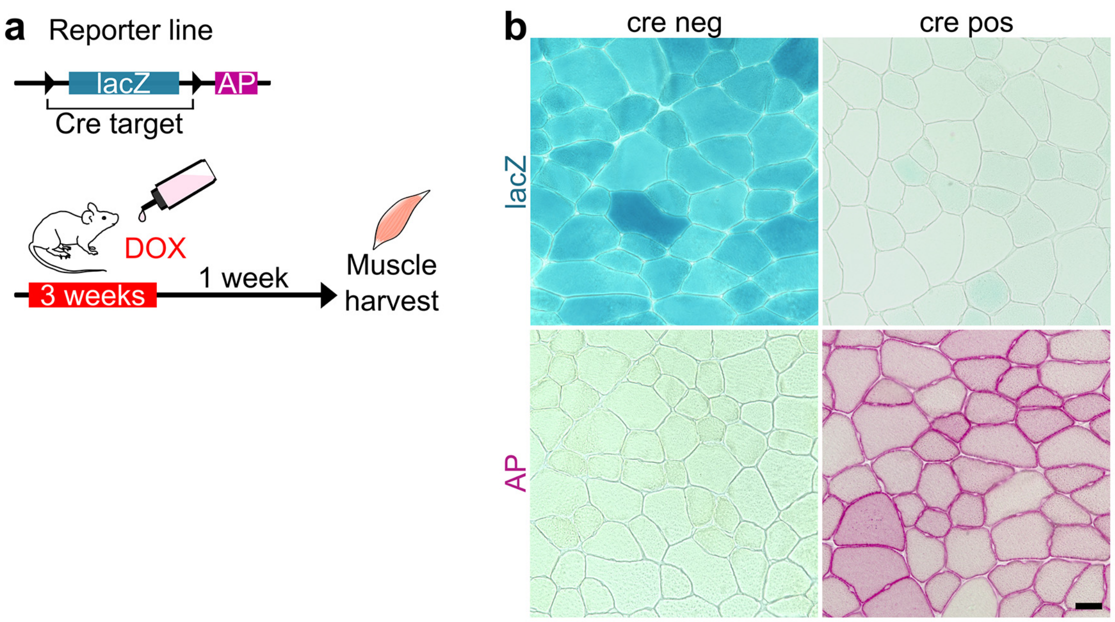

Supplementary figure 10: Efficient Cre-recombination in adult muscle fibers.

a-b Schematic representation of the experimental outline. *Z/AP* reporter mice were mated to *HSA-rtTA/TRE-Cre*$^{+/-}$ mice (**a**) to analyse the efficiency of recombination. LacZ and AP staining confirm complete recombination of the reporter locus by *HSA-rtTA/TRE-Cre* (n=2) in adult muscle fibers after doxycycline (DOX) treatment (**b**) (n = 1 control, scale bar: 50 µm).

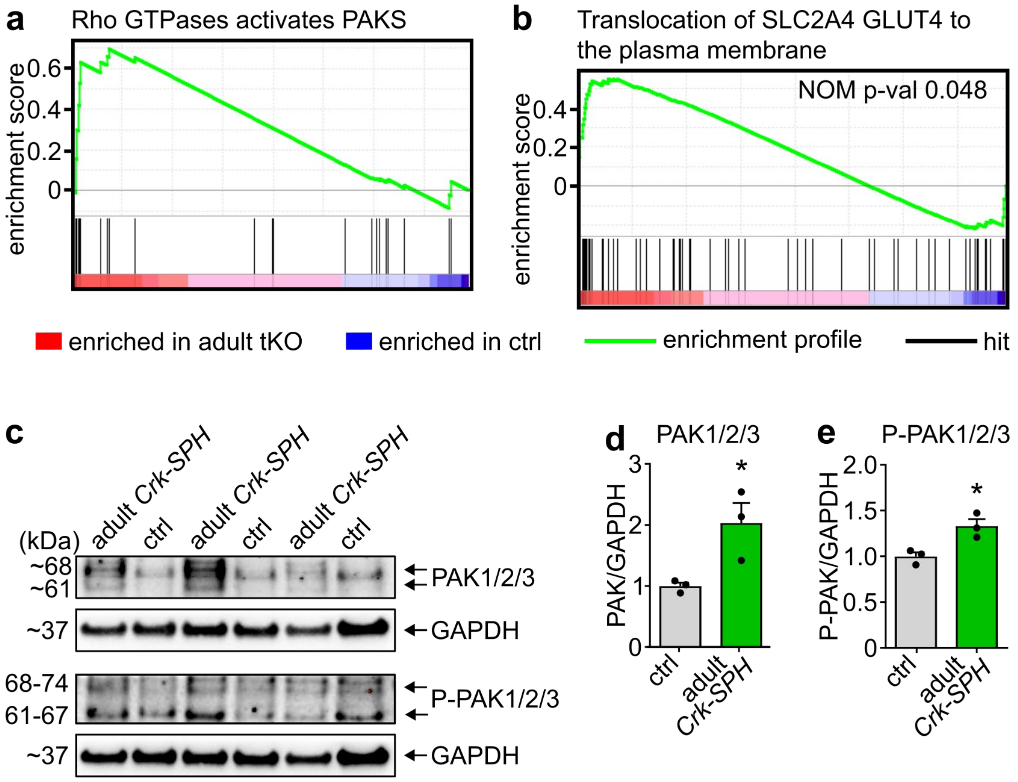

Supplementary figure 11: Activation of RAC-GTP downstream targets in adult *miR-1/206/133* tKO and adult CRK overexpressing muscles

a-b GSEA using transcriptome data of adult *miR-1/206/133* tKO (n = 3) vs. control TA muscles (n = 2, control = *miR-1-1/133a-2*$^{-/-}$*//miR-1-2/133a-1*$^{lox/lox}$*//miR-206/133b*$^{-/-}$*//HSA-rtTA-TRE- Cre*$^{+/+}$, nominal p value (NOM p-val), p = 0.048 [male, ~44 weeks]).

c-e Western blot analysis of increased PAK1/2/3 expression and phosphorylation in adult *Crk-SPH* (n = 3) compared to control mice (n = 3). GAPDH served as loading control. (control = *CAG-LSL-dCas9-SPH*$^{+/+}$, Mann-Whitney U test, one-tailed, *p =0.05, Data are presented as mean +/- SEM [male, female, ~14 weeks]). Source data are provided as a Source Data file.

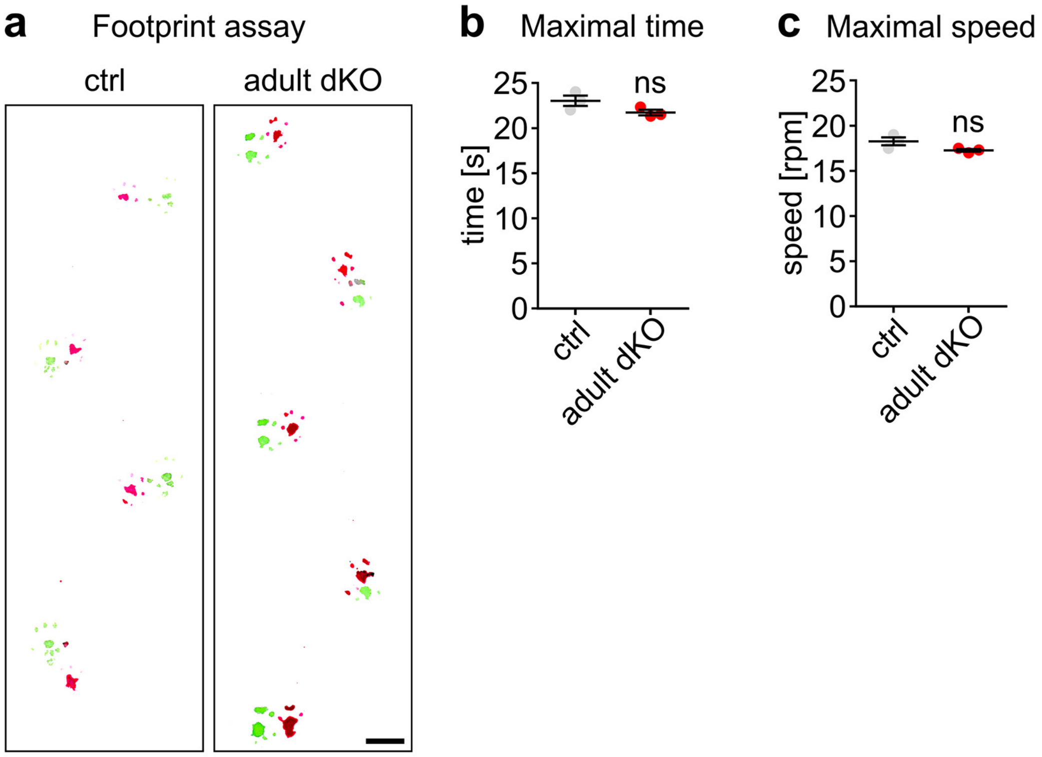

Supplementary figure 12: Motor coordination is impaired in 44-weeks old *miR-1/206/133* tKO, but not in *miR-1/133a* dKO animals.

a Footprint assays demonstrate absence of motor coordination impairment in adult *miR-1/133a* dKO (n=3) mice (n= 3, control = *miR-1-1/133a-2*$^{+/+}$*//miR-1-2/133a-1*$^{lox/lox}$*//miR-206/133b*$^{+/+}$*//Pax7-Cre*$^{+/+}$ [male, ~44 weeks]). Scale bar: 1 cm.

b-c Rotarod analysis demonstrates normal motor functions in adult *miR-1/133a* dKO (n=3) (n= 3, control = *miR-1-1/133a-2*$^{+/+}$*//miR-1-2/133a-1*$^{lox/lox}$*//miR-206/133b*$^{+/+}$*//Pax7-Cre*$^{+/+}$, Mann-Whitney U test, one-tailed, ns = not significant, Data are presented as mean values +/- SEM [male, ~44 weeks]). Source data are provided as a Source Data file.

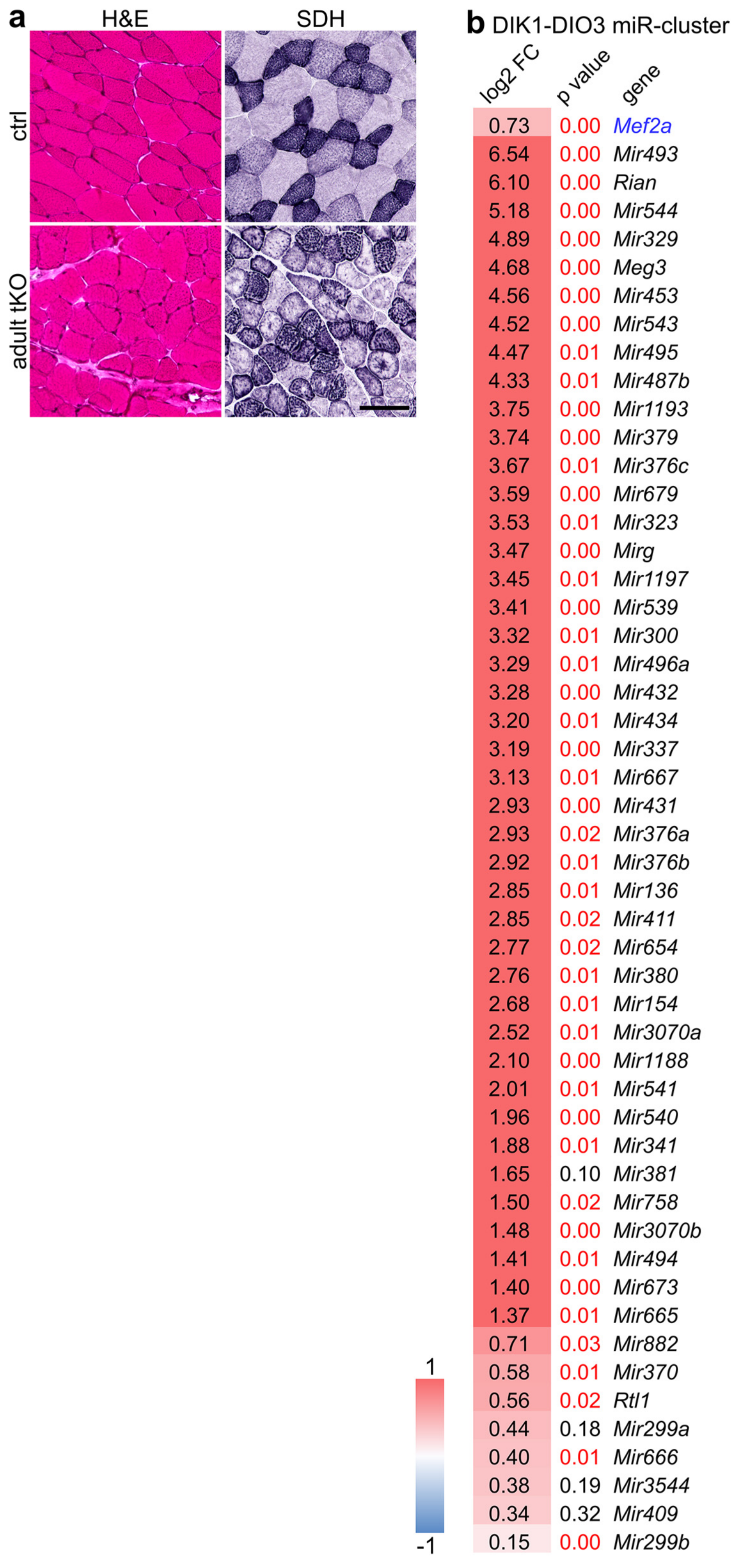

Supplementary figure 13: Upregulation of the *Dlk1-Dio3* microRNA mega-cluster and mitochondrial dysfunction in adult *miR-1/206/133* tKO muscles.

a Histological examination of adult *miR-1/206/133* tKO (n = 3, lower row) and control mice (n = 3, upper row). (**a**) H&E (left) and SDH staining (right) of TA muscles (control: *miR-1-1/133a-2*$^{-/-}$*//miR-1-2/133a-1*$^{lox/lox}$*//miR-206/133b*$^{-/-}$*//HSA-rtTA-TRE-Cre*$^{+/+}$, scale bar: 50 µm [male, ~44 weeks]).

b Expression analysis of genes in the *Dlk1-Dio3* cluster and of *Mef2a* (blue) in adult *miR-1/206/133* tKO (n = 3) muscles (log_2 FC = Fold Change log_2, n = 2, control = *miR-1-1/133a-2*$^{-/-}$*//miR-1-2/133a-1*$^{lox/lox}$*//miR-206/133b*$^{-/-}$*//HSA-rtTA-TRE-Cre*$^{+/+}$, modified t-test, red = significant) [male, ~44 weeks.

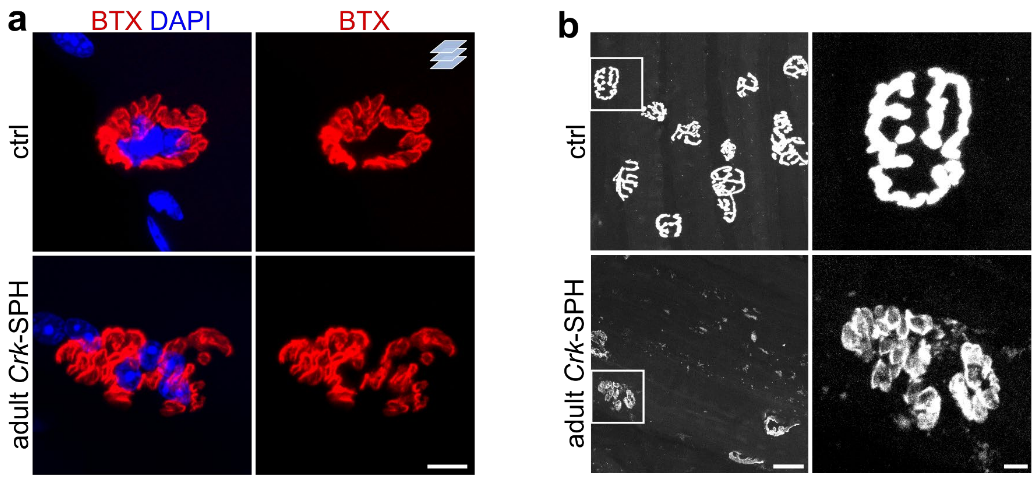

Supplementary figure 14: Impaired maintenance of NMJ integrity after skeletal muscle-specific overexpression of CRK.

a BTX staining (red) of isolated single myofibers from *flexor digitorum brevis* muscles of adult *Crk-SPH* mice (n = 3) in comparison to controls (n = 3, control of *Crk-SPH* = *HSA-rtTA-TRE-Cre*$^{+/+}$ or *CAG-LSL-dCas9-SPH*$^{+/+}$ [~14 weeks, male]). DAPI staining in blue. Z-stacks (scale-bar: 20 µm).

b Whole mount BTX staining of EDL (*extensor digitorum longus*) muscles from adult *Crk-SPH* mice (n = 2) in comparison to controls (n = 2, control of *Crk-SPH* = *HSA-rtTA-TRE-Cre*$^{+/+}$ or *CAG-LSL-dCas9-SPH*$^{+/+}$[~14 weeks, male]) scale-bar: 20 µm (left), 5 µm (right).

References

1. Billing AM, Ben Hamidane H, Graumann J. Quantitative proteomic approaches in mouse: stable isotope incorporation by metabolic (SILAC) or chemical labeling (reductive dimethylation) combined with high-resolution mass spectrometry. *Curr Protoc Mouse Biol* **5**, 1-20 (2015).

2. Boersema PJ, Raijmakers R, Lemeer S, Mohammed S, Heck AJ. Multiplex peptide stable isotope dimethyl labeling for quantitative proteomics. *Nat Protoc* **4**, 484-494 (2009).

3. Rappsilber J, Ishihama, Y., Mann, M. . Stop and go extraction tips for matrix-assisted laser desorption/ionization, nanoelectrospray, and LC/MS sample pretreatment in proteomics. *Analytical chemistry* **75(3)**, 663-670 (2003).

4. Shevchenko A, Tomas H, Havlis J, Olsen JV, Mann M. In-gel digestion for mass spectrometric characterization of proteins and proteomes. *Nat Protoc* **1**, 2856-2860 (2006).

5. Kiweler M, Looso M, Graumann J. MARMoSET - Extracting Publication-ready Mass Spectrometry Metadata from RAW Files. *Mol Cell Proteomics* **18**, 1700-1702 (2019).

6. Cox J, Mann M. MaxQuant enables high peptide identification rates, individualized p.p.b.-range mass accuracies and proteome-wide protein quantification. *Nat Biotechnol* **26**, 1367-1372 (2008).

7. Cox J, Neuhauser N, Michalski A, Scheltema RA, Olsen JV, Mann M. Andromeda: a peptide search engine integrated into the MaxQuant environment. *J Proteome Res* **10**, 1794-1805 (2011).

8. Cox J, Hein MY, Luber CA, Paron I, Nagaraj N, Mann M. Accurate proteome-wide label-free quantification by delayed normalization and maximal peptide ratio extraction, termed MaxLFQ. *Mol Cell Proteomics* **13**, 2513-2526 (2014).

9. UniProt C. UniProt: a worldwide hub of protein knowledge. *Nucleic Acids Res* **47**, D506-D515 (2019).

10. Ritchie ME*, et al.* limma powers differential expression analyses for RNA-sequencing and microarray studies. *Nucleic Acids Res* **43**, e47 (2015).
