# Supplementary material for: Control of CRK-RAC1 activity by the miR-1/206/133 miRNA family is essential for neuromuscular junction function
Source: Nat Commun. 2022 Jun 8;13:3180. doi: 10.1038/s41467-022-30778-7 (PMC9178026; doi:10.1038/s41467-022-30778-7)
Supplement: Supplementary file 3 — Description of Additional Supplementary Files [file 41467_2022_30778_MOESM3_ESM.pdf]

Description of Additional Supplementary Information File

Supplementary Data 1: LC-MS/MS Parametrization. Description of all LC-MS/MS parameters, chromatography gradients and the parametrization of the MaxQuant suite of algorithms.
