# Supplementary material for: Control of CRK-RAC1 activity by the miR-1/206/133 miRNA family is essential for neuromuscular junction function
Source: Nat Commun. 2022 Jun 8;13:3180. doi: 10.1038/s41467-022-30778-7 (PMC9178026; doi:10.1038/s41467-022-30778-7)
Supplement: Supplementary file 5 — Reporting Summary [file 41467_2022_30778_MOESM5_ESM.pdf]

nature portfolio

Corresponding author(s): Thomas Boettger&Thomas Braun

Last updated by author(s): Apr 1, 2022

Reporting Summary

Nature Portfolio wishes to improve the reproducibility of the work that we publish. This form provides structure for consistency and transparency in reporting. For further information on Nature Portfolio policies, see our Editorial Policies and the Editorial Policy Checklist.

Statistics

For all statistical analyses, confirm that the following items are present in the figure legend, table legend, main text, or Methods section.

n/a	Confirmed	
☐	☒	The exact sample size (*n*) for each experimental group/condition, given as a discrete number and unit of measurement
☐	☒	A statement on whether measurements were taken from distinct samples or whether the same sample was measured repeatedly
☐	☒	The statistical test(s) used AND whether they are one- or two-sided<br>*Only common tests should be described solely by name; describe more complex techniques in the Methods section.*
☒	☐	A description of all covariates tested
☐	☒	A description of any assumptions or corrections, such as tests of normality and adjustment for multiple comparisons
☐	☒	A full description of the statistical parameters including central tendency (e.g. means) or other basic estimates (e.g. regression coefficient) AND variation (e.g. standard deviation) or associated estimates of uncertainty (e.g. confidence intervals)
☐	☒	For null hypothesis testing, the test statistic (e.g. *F*, *t*, *r*) with confidence intervals, effect sizes, degrees of freedom and *P* value noted<br>*Give P values as exact values whenever suitable.*
☒	☐	For Bayesian analysis, information on the choice of priors and Markov chain Monte Carlo settings
☒	☐	For hierarchical and complex designs, identification of the appropriate level for tests and full reporting of outcomes
☒	☐	Estimates of effect sizes (e.g. Cohen's *d*, Pearson's *r*), indicating how they were calculated

Our web collection on statistics for biologists contains articles on many of the points above.

Software and code

Policy information about availability of computer code

Data collection	*Provide a description of all commercial, open source and custom code used to collect the data in this study, specifying the version used OR state that no software was used.*
Data analysis	BD FACS Diva v8 Software, StepOne Software v2.3, ImageJ 1.53k, GraphPad PRISM 6.07, GSEA v3.0, IMARIS x64 9.6.0, IncuCyte ZOOM 2016A, Zen 2.1, MaxQuant suite of algorithms (proteome: v. 1.6.0.1; interactome: v. 1.6.6.0)

For manuscripts utilizing custom algorithms or software that are central to the research but not yet described in published literature, software must be made available to editors and reviewers. We strongly encourage code deposition in a community repository (e.g. GitHub). See the Nature Portfolio guidelines for submitting code & software for further information.

Data

Policy information about availability of data

All manuscripts must include a data availability statement. This statement should provide the following information, where applicable:

- Accession codes, unique identifiers, or web links for publicly available datasets
- A description of any restrictions on data availability
- For clinical datasets or third party data, please ensure that the statement adheres to our policy

Data have been deposited in public data bases. Microarray data are available at Arrayexpress (E-MTAB-10229; http://www.ebi.ac.uk/arrayexpress/experiments/E-MTAB-10229; E-MTAB-10228; http://www.ebi.ac.uk/arrayexpress/experiments/E-MTAB-10228, ; http://www.ebi.ac.uk/arrayexpress/experiments/E-MTAB-10227). Proteome data are available at ProteomeCentral (http://proteomecentral.proteomexchange.org/cgi/GetDataset?ID=PXD032882) with the dataset identifier (JPST001553) PXD032882. Source data are provided as a Source Data files.

Field-specific reporting

Please select the one below that is the best fit for your research. If you are not sure, read the appropriate sections before making your selection.

[x] Life sciences [ ] Behavioural & social sciences [ ] Ecological, evolutionary & environmental sciences

For a reference copy of the document with all sections, see nature.com/documents/nr-reporting-summary-flat.pdf

Life sciences study design

All studies must disclose on these points even when the disclosure is negative.

Sample size	Sample size were determined based on stablished practice and applicable standards. We opted for sample sizes which are routinely employed sample sizes in the field.<br>Each experiment in which data were quantified was performed with at least 3 replicates. For in vivo and in vitro studies, the numbers of biological replicates are indicated in the figure legends or the method part. For In vitro studies, experiments in which data were not quantified were performed with at least two replicates.
Data exclusions	No data were excluded.
Replication	The experiments in this study were all replicated and reproduced as indicated. All in vivo studies were performed with indicated number of animals. Sample sizes and statistical analyses and significance levels are all indicated in the figure legends or the method part. All physiological tests were repeated at least two times per tested individual.
Randomization	Experimental animals were randomized for their allocation in groups. All animals were numbered and experiments were performed if possible in a blinded pattern or genotyped after the performed experiment. After data collection, genotypes were revealed and animals assigned to groups for analysis.
Blinding	Blinding was performed for the in vivo experiments. The experimenter did not know which mice belonged to which experimental group during data collection. In vitro experiments were not blinded during analysis because treatment, collection, or analysis of the material required labeling of the different samples. Positive controls, negative controls and samples were treated and analyzed in exactly the same manner.

Reporting for specific materials, systems and methods

We require information from authors about some types of materials, experimental systems and methods used in many studies. Here, indicate whether each material, system or method listed is relevant to your study. If you are not sure if a list item applies to your research, read the appropriate section before selecting a response.

Materials & experimental systems

n/a	Involved in the study	
[ ]	[x]	Antibodies
[ ]	[x]	Eukaryotic cell lines
[x]	[ ]	Palaeontology and archaeology
[ ]	[x]	Animals and other organisms
[x]	[ ]	Human research participants
[x]	[ ]	Clinical data
[x]	[ ]	Dual use research of concern

Methods

n/a	Involved in the study	
[x]	[ ]	ChIP-seq
[ ]	[x]	Flow cytometry
[x]	[ ]	MRI-based neuroimaging

Antibodies

Antibodies used

Bungarotoxin Alexa Fluor555 conjugate (1:300, ThermoFisher #B35451)
Bungarotoxin Alexa Fluor594 conjugate (1:300, ThermoFisher #B13423)
Wheat germ agglutinin Alexa Fluor 488 Conjugate (1:300, ThermoFisher #W11261)
anti-CRK (4 µg for IP, 1:1000 for PLA and Western Blot, Santa Cruz #sc-390132, B4)
anti-HA-tag (1:1000, Covance #MMS-101P, 16B12)
anit-RAC1 (1:1000, part of kit: ThermoFisher #16118)
anti-RALA (1:1000, BD Bioscience s#610222, Clone 8)
anti-Laminin (1:1000, Sigma #L9393)
anti-CRK (1:1000, BD Biosciences #610035, 22/Crk)
anti-GFP (1:1000, ThermoFisher #A-11122)
goat anti-mouse IgG-Alexa Fluor488 (1:1000, Jackson #115-545-205)
goat anti-mouse IgG-Alexa Fluor594 (1:1000, Jackson #115-585-205)
goat anti-rabbit IgG-Alexa488 (1:1000, ThermoFischer #A11070)

anti-mouse-HRP (1:5000, ThermoFischer #31450)
anti-rabbit-HRP (1:5000, ThermoFischer #31460)
anti-CD45-APC (1:100, eBioscience #17-0451, 30-F11)
anti-CD31-APC (1:100, eBioscience #17-0311, 390)
anti-Ly-6A/E (Sca-1)-APC (1:100, eBioscience #17-5981, D7)
anti-CD34-Alexa450 (1:100, eBioscience #48-0341-82, RAM34)
anti-Integrin-FITC (1:100, MBL #K0046-4, 3C12)
anti-FARP1 (1:5000, ThermoFisher #PA5-99105)
anti-MEF2A (1:1000, CST #9736)
anti-GAPDH (1:1000, CST #2118, 14C10)
PAK1/2/3 (1:1000, CST #2604)
Phospho-PAK1/2/3 (1:1000, CST #2606)
anti-SV2 (1:200, synaptic vesicles, DSHB #SV2)
anti-neurofilament (1:500, BioLegend #837904, SMI 312)

Validation

Bungarotoxin Alexa Fluor555 conjugate (ThermoFisher #B35451) manufacture's website: a 74-amino acid peptide extracted from Bungarus multicinctus venom, binds with high affinity to the !-subunit of the nicotinic acetylcholine receptor (AChR) of neuromuscular junctions, validated in DOI: 10.1194/jlr.M600182-JLR200; !-Bungarotoxin Alexa Fluor594 conjugate (ThermoFisher #B13423) manufacture's website: a 74-amino acid peptide extracted from Bungarus multicinctus venom, binds with high affinity to the !-subunit of the nicotinic acetylcholine receptor (AChR) of neuromuscular junctions, validated in DOI: 10.1038/nn801; Wheat germ agglutinin Alexa Fluor 488 Conjugate (ThermoFisher #W11261) manufacture's website: binds to sialic acid and Nacetylglucosaminyl residues, validated in DOI: 10.1152/ajpgi.00209.2012; anti-CRK (BD Biosciences #610035 (Clone 22/Crk) manufacture's website: recommended for Western blot analysis and Immunofluorescent staining, validated DOI: 10.1083/jcb.200111032; anti-CRK (Santa Cruz #sc-390132) manufacture's website: recommended for Western Blotting, immunoprecipitation, immunofluorescence, validated byWestern Blot analysis with whole muscle lysate and in DOI: https://doi.org/10.4049/jimmunol.1701639; anti-HA-tag (Covance #MMS-101P) validated by Western Blot analysis with whole muscle lysates of HA-tagged dCas9 expressing mice from DOI: 10.1038/s41593-017-0060-6; anit-RAC1 (part of kit: ThermoFisher #16118) validated by GTP"S and GDP treatment of cell lysates; anti-RALA (BD Bioscience s#610222) validated by Western Blot analysis with whole muscle lysates; anti-Laminin (Sigma #L9393) validated with immunofluorescence staining of muscle cross sections and in DOI: https://doi.org/10.15252/embj.2020105098; anti-CRK (BD Biosciences #610035) validated by immunofluorescence staining of muscle cross sections and manufacture's website: validation using Western Blot analysis of Crk on a HeLa lysate; anti-GFP (ThermoFisher #A-11122) manufacture's website: Advanced Verification: This Antibody was verified by Relative expression to ensure that the antibody binds to the antigen stated, additionally the manufacturer tested this antibody via Immunofluorescent analysis of a GFP Tag; goat anti-mouse IgG-Alexa Fluor488 (Jackson #115-545-205); goat anti-mouse IgG-Alexa Fluor594 (Jackson #115-585-205) validated by immunofluorescence staining of muscle cross sections; goat anti-rabbit IgG-Alexa488 (ThermoFischer #A11070) validated by immunofluorescence staining of muscle cross sections; anti-mouse-HRP (ThermoFischer #31450) manufacture's website: Product #31450 has been successfully used in Western blot, and ICC applications, Antibody Specificity: This antibody reacts with the heavy chains of mouse IgG and with the light chains common to most mouse immunoglobulins, validated by Western Blot analysis with whole muscle lysate; anti-rabbit-HRP (ThermoFischer #31460) manufacture's website: Product # 31460 has been successfully used in Western blot, IHC and IP applications, Product # 31460 reacts with the heavy chains of rabbit IgG and with the light chains common to most rabbit immunoglobulins, but does not react against non-immunoglobulin serum proteins, validated by Western Blot analysis with whole muscle lysate; anti-CD45-APC (eBioscience #17-0451), anti-CD31-APC (eBioscience #17-0311), anti-Ly-6A/E (Sca-1)-APC (eBioscience #17-5981), anti-CD34-Alexa450 (eBioscience #48-0341-82) and anti-Integrin-FITC (MBL #K0046-4) validated by flow cytometric analysis by DOI: 10.15252/embj.2020105098; anti-FARP1 (ThermoFisher #PA5-99105) manufacture's website: recommended for Western Blot, Immunohistochemistry, Immunocytochemistry, ELISA, Validated byWestern Blot using whole muscle lysate; anti-MEF2A (CST #9736) validated in DOI: 10.1016/j.cmet.2018.02.022; anti-GAPDH (CST #2118) validated in DOI: 10.1126/sciadv.abi6648; PAK1/2/3 (CST #2604) manufacture's website: recommended for western blot, DOI: 10.1093/hmg/ddab277 and validated by western blot using whole muscle lysate; Phospho-PAK1/2/3 (CST #2606) manufacture's website: recommended for western blot, validated by DOI: 10.1093/hmg/ddab277 and by western blot using whole muscle lysate; anti-SV2 (DSHB #SV2) manufacture's website: recommended for immunofluorescence staining, validated in DOI: 10.1371/journal.pone.0091643 and DOI: 10.1002/cne.23917; anti-neurofilament (BioLegend #837904) manufacture's website: recommended for immunofluorescence staining, validated in DOI: 10.1101/gad.1685008 and DOI: 10.1016/s0002-9440(10)63360-3

Eukaryotic cell lines

Policy information about cell lines

Cell line source(s)	C2C12 (C2 cells) source(s) ATCC #CRL-1772
Authentication	Cell lines were used as obtained from the source. No further authentication was done.
Mycoplasma contamination	The C2C12 cell line was tested for absence of mycoplasma contamination (Information on the manufacturer's website)
Commonly misidentified lines (See ICLAC register)	No commonly misidentified cell lines were used.

Animals and other organisms

Policy information about studies involving animals; ARRIVE guidelines recommended for reporting animal research

Laboratory animals	Laboratory animals: Mus musculus

ctKO: (E10.5, sex not defined) miR-1-1/miR-133a-2-/-//miR-1-2/miR-133a-1-/-//miR-206/133b-/-

sKO: (E18.5, P21, sex not defined) miR-206/133b-/-

dKO: (E18.5, P21, sex not defined// adult: 14-44 weeks male and female) miR-1-1/miR-133a-2-/-//miR-1-2/miR-133a-1lox/lox//Pax7-Cre+/-

tKO: (E18.5, sex not defined) miR-1-1/miR-133a-2-/-//miR-1-2/miR-133a-1lox/lox//miR-206/133b-/-//Pax7-Cre+/-

adult tKO: (15-44 weeks, male and female) miR-1-1/miR-133a-2-/-//miR-1-2/miR-133a-1lox/lox//miR-206/133b-/-//HSA-rtTA-TRE-Cre+/-

Crk-tg: (E18.5, sex not defined) Crk-tg+/-

Crk-SPH: (E18.5, sex not defined) sgRNA+/-//Pax7-Cre+/-//CAG-LSL-dCas9-SunTag-p65-HSF1+/-

adult Crk-SPH: (10-16 weeks, male and female) sgRNA+/-// HSA-rtTA/TRE-Cre +/-//CAG-LSL-dCas9-SunTag-p65-HSF1+/-

Species, strain, sex, age and genetic modifications are reported in the manuscript.

Wild animals: Studies did not involve wild animals.

Field-collected samples: Studies did not involve samples collected in the field.

Ethics oversight: All animal experiments were done in accordance with the Guide for the Care and Use of Laboratory Animals published by the US National Institutes of Health (NIH Publication No. 85-23, revised 1996) and were approved by the responsible Committee for Animal Rights Protection of the State of Hessen (Regierungspraesidium Darmstadt, Wilhelminenstr. 1-3, 64283 Darmstadt, Germany) with the project number B2/1054 and B2/1234.

Note that full information on the approval of the study protocol must also be provided in the manuscript.

Flow Cytometry

Plots

Confirm that:

- [x] The axis labels state the marker and fluorochrome used (e.g. CD4-FITC).
- [x] The axis scales are clearly visible. Include numbers along axes only for bottom left plot of group (a 'group' is an analysis of identical markers).
- [x] All plots are contour plots with outliers or pseudocolor plots.
- [x] A numerical value for number of cells or percentage (with statistics) is provided.

Methodology

Sample preparation: Skeletal muscles tissue was collected from individual animals in DMEM (Merck #D5796) containing 2% Penicilin-Streptamycin. Next, the tissue was chopped and digested using Dispase (BD #354-235; 1:10 in solution) and Collagenase II (Worthington #LS004185; 1:10 in solution) for 30 min each. The suspensions were treated with fetal calf serum and filtered through 100 µm, 70 µm and 40 µm cell easystainers. Remaining cells were resuspended after centrifugation and incubated with the following combination of antibodies, anti-CD45-APC, anti-CD31-APC and anti-Ly-6A/E (Sca-1)-APC and anti-CD34-Alexa450 (eBioscience #17-0451, 17-0311, 17-5981, #48-0341-82; each 1:100) and anti-integrin-FITC (MBL #K0046-4; 1:100) for 30 min at 4 °C. Afterwards, the cells were washed, resuspended and incubated with anti-APC MicroBeads (Miltenyi #130-090-855) for 20 min at 4 °C. All CD31, CD45 and Sca-1 positive cells were depleted using the Milteny AutoMACS. The remaining cells were treated with DAPI (Invitrogen, #D1306), to identify dead cells during sorting with a FACS (Aria III, BD FACS Diva v8 Software).

Instrument: Aria III, BD

Software: FACS Diva v8 Software, BD

Cell population abundance: Pre-enriched, single and living MuSCs cells were detectd by Integrin a7 – FITC staining. Pre-enriched fraction was used to sort Integrin-a7 FITC positive MuSC population. MuSCs population was defined using FMO (Fluorescense Minus One) FITC control (not treated with Integrin a7 – FITC). CD31-APC, CD45-APC and SCA1-APC population were depleted and dead cells were excluded via DAPI staining. The MuSC abundance in the pre-enriched fraction was 9.6% of all detected cells.

Gating strategy	Gating strategy identifying cells, singlets, using pre-enriched and live cells to detect MuSCs positive for Integrin a7 – FITC. CD31-APC, CD45-APC and SCA1-APC population were depleted using magnetic columns on AutoMACS (Miltenyi), and dead cells were excluded using DAPI staining. Pre-enriched fraction was used to sort Integrin-a7 FITC positive MuSC population. APC = allophycocyanin; FITC = fluorescein isothiocyanate; PE = phycoerythrin.

☒ Tick this box to confirm that a figure exemplifying the gating strategy is provided in the Supplementary Information.
